# Towards reliable diagnostics of prostate cancer via breath

**DOI:** 10.1038/s41598-021-96845-z

**Published:** 2021-09-15

**Authors:** K. S. Maiti, E. Fill, F. Strittmatter, Y. Volz, R. Sroka, A. Apolonski

**Affiliations:** 1 Lehrstuhl für Experimental Physik, Ludwig-Maximilians-Universität München, Am Coulombwall 1, 85748 Garching, Germany; 2 Max-Planck-Institut für Quantenoptik, Hans-Kopfermann-Strasse 1, 85748 Garching, Germany; 3 Urologische Klinik und Poliklinik des Klinikums der Ludwig-Maximilians-Universität München in Grosshadern, 81377 Munich, Germany; 4 Laser-Forschungslabor, LIFE Center, University Hospital, LMU Munich, 82152 Planegg, Germany; 5 Novosibirsk State University, 630090 Novosibirsk, Russia; 6 Institute of Automation and Electrometry SB RAS, 630090 Novosibirsk, Russia

**Keywords:** Cancer, Optics and photonics

## Abstract

Early detection of cancer is a key ingredient for saving many lives. Unfortunately, cancers of the urogenital system are difficult to detect at early stage. The existing noninvasive diagnostics of prostate cancer (PCa) suffer from low accuracy (< 70%) even at advanced stages. In an attempt to improve the accuracy, a small breath study of 63 volunteers representing three groups: (1) of 19 healthy, (2) 28 with PCa, (3) with 8 kidney cancer (KC) and 8 bladder cancer (BC) was performed. Ultrabroadband mid-infrared Fourier absorption spectroscopy revealed eight spectral ranges (SRs) that differentiate the groups. The resulting accuracies of supervised analyses exceeded 95% for four SRs in distinguishing (1) vs (2), three for (1) vs (3) and four SRs for (1) vs (2) + (3). The SRs were then attributed to volatile metabolites. Their origin and involvement in urogenital carcinogenesis are discussed.

## Introduction

In Germany, cancers of the urogenital tract demonstrate significant rates: prostate (PCa; the list of abbreviations—just before the section “References”) has 10.3% of all new cancers, bladder (BC)—5.9% and kidney (KC)—2.7%. In 2019, the death ranks were 3 for PCa, 8 for BC and 11 for KC. PCa represents the most common malignant tumor in Western men, with 30% in their third decade, 50% in their fifth decade and over 75% in their eighth decade^1^. Similar aging trends were observed for KC and BC^2^. So far, reliable diagnostics and prognostics of PCa do not exist^3^. Among general techniques, magnetic resonance imaging demonstrates high enough detection rate but only at late cancer stage. Ultrasound imaging of prostate capsule artery and urethral artery does not show strong correlation with the risk of PCa^4^. Therefore, biomarker-based detection techniques are now in the focus of research groups. The existing liquid-phase biomarker serum prostate-specific antigen (PSA) demonstrates 70% sensitivity and 60% specificity^5^, well below the desirable accuracy beyond 95%. Therefore new, more general biomarkers like exosomes or early prostate cancer antigen are now in the test phase. Urine headspace was already demonstrated as a source of gas-phase biomarkers for detecting diseases of the urogenital system, especially the prostate. For example, a combination of PSA and urinary biomarkers revealed 74% accuracy^6^ for PCa. A recent study based on a panel of 6 urine biomarkers in gas phase resulted in 86% detection accuracy^7^. With the help of dogs, the accuracy of urine headspace analysis achieved 95%^8^. Nevertheless, animal models are not reliable “tools” of the twenty-first century, with rather low reproducibility daily. All these achievements manifest significant progress in the last decade, though the experimental results do not meet clinicians’ expectations. The BC detection includes several methods, with the best accuracies of 97% achieved for non-invasive liquid urine markers and invasive cystoscopy^9^. The KC detection demonstrates 99% accuracy across all stages by using plasma cell-free DNA methylomes^10^, though so far no clinically validated noninvasive biomarkers revealed.

Several experimental tools are available or will become available soon for detecting cancer-sensitive metabolites in gas phase. They include mass spectrometry, electronic nose and to a lesser extent, optical (mid-infrared) spectroscopy and near infrared photoacoustic spectroscopy. Mass spectrometry (MS) in combination with gas chromatography (GC) is a powerful technique, though suffering from several practical and technical limitations^11,12^. Moreover, this tool is costly and needs essential preprocessing. Electronic nose cannot be used for molecular identification and quantification^13^, although this tool is attractive because of small size. Both tools were already applied for studying lung^14^ and breast^15^ cancers via breath, as well as BC^6^ and PCa^7^ via metabolites in urine headspace. Up to now, these tools as well as photoacoustic spectroscopy^16^ do not allow reaching the desirable detection accuracy.

In this study, we used the third mentioned technology, namely mid-infrared spectroscopy applied to breath. Its advantage is based on the fact that all biological molecules have unique absorption spectra (called fingerprints) in the mid-infrared range 2.5–20 µm (or 500–4000 cm^−1^). Moreover, gas-phase spectroscopy offers a possibility to resolve and reliably identify small metabolites (see the “Discussion” section). Its reliability is based on simple preprocessing and processing steps. However, spectroscopy is far from the advanced state of GC–MS^17^. There are two main reasons for that. First, the high water concentration in breath or urine headspace samples results in broad and strong absorption bands making detection of trace molecules difficult. Second, the absence of powerful (i.e. laser-based) mid-infrared sources covering the fingerprint range. We are witnessing significant progress in this direction regarding the signal-to-noise ratio of the measured spectra behind the biological samples^18,19^. However, the spectral coverage is still not satisfactory (for illustration, see Table 1). As a result, only tens of metabolites have been identified so far in real bio-samples.

**Table 1:** SRs where average (over different cancer groups) absorption spectra differ from the healthy group. Columns 5–6: results of unsupervised PCA + ANOVA, columns 7–9: results of supervised k-fold validation for different cancer groups.

The center of SR, cm^−1^	Identified metabolite	Metabolite molecular mass, amu	Concentration for healthy in ppb/ the concentration ratio of cancer to healthy	p-values for PC1/PC2/PC3/PC4 analyses; healthy vs cancer	Variance of PC1/PC2/PC3/PC4 in %	Ninefold validation: accuracy/sensitivity/specificity/error (SD); healthy vs PCa	Ninefold validation: accuracy/sensitivity/specificity/error (SD); healthy vs PCa + BC + KC	Sevenfold validation: accuracy/sensitivity/specificity/error (SD); healthy vs BC + KC
1005	Acetic anhydride	102.09	83, 2.1	1 × 10^–4^/3 × 10^–1^/ 5 × 10^–6^/4 × 10^–1^	45/9/ 7/ 6	0.98/0.99/0.97/0.02	0.98/0.99/0.97/0.02	0.95/0.94/0.96/0.04
1190	Propyl propionate	116.16	87, 1.4	4 × 10^–1^/6 × 10^–4^/ 3 × 10^–2^/6 × 10^–1^	37/18/10/7	0.97/0.97/0.97/0.02	0.97/0.97/0.97/0.02	0.97/0.98/0.96/0.03
1203	Ethyl vinyl ketone	84.12	130, 0.8	4 × 10^–1^/6 × 10^–4^/ 3 × 10^–2^/6 × 10^–1^	37/18/10/7	0.97/0.97/0.97/0.02	0.97/0.97/0.97/0.02	0.97/0.98/0.96/0.03
530	Acetaldehyde	44.05	690, 1.4	3 × 10^–4^/4 × 10^–2^/ 6 × 10^–2^/2 × 10^–1^	76/6/2/1	0.83/0.95/0.66/0.02	0.83/0.95/0.66/0.02	0.94/1.0/0.85/0.02
1050	Carbon dioxide	44.01	4 × 10^7^, 0.8	3 × 10^–1^ /6 × 10^–2^/ 1 × 10^–2^/ 1 × 10^–2^	88/6/2/1	0.92/0.97/0.86/0.02	0.92/0.97/0.86/0.02	0.87/0.87/0.87/0.02
2170	Carbon monoxide	28.01	2 × 10^3^, 1.1	9 × 10^–1^ /1 × 10^–1^/ 1 × 10^–2^/4 × 10^–2^	88/2/2/1	0.79/0.88/0.68/0.07	0.79/0.88/0.68/0.04	0.81/0.68/0.92/0.03
1130	Ethyl pyruvate	116.11	183, 0.8	5 × 10^–1^/5 × 10^–1^/ 9 × 10^–1^/1 × 10^–1^	26/17/9/9	0.99/0.99/0.99/0.02	0.99/0.99/0.99/0.02	0.95/0.93/0.96/0.04
1170	Methyl butyrate	102.13	100, 1.4	3 × 10^–1^/9 × 10^–1^/ 2 × 10^–1^/6 × 10^–2^	28/17/10/9	0.98/0.98/0.98/0.02	0.98/0.99/0.98/0.02	0.94/0.93/0.96/0.04

Using laser-free broadband mid-infrared Fourier-transform spectroscopy applied to breath, we recently identified derivatives of short-chain fatty acids (SCFAs) in the breath of patients with cerebral palsy. These metabolites allowed us to detect the disease diagnosis’s detection accuracy > 90%^20^. It was the first demonstration of the power of gas spectroscopy applied to complex movement disorder related to the brain damage. Here, we demonstrate that such spectroscopy provides specific information about PCa and two other urogenital cancers.

## Results

**Figure 1:**
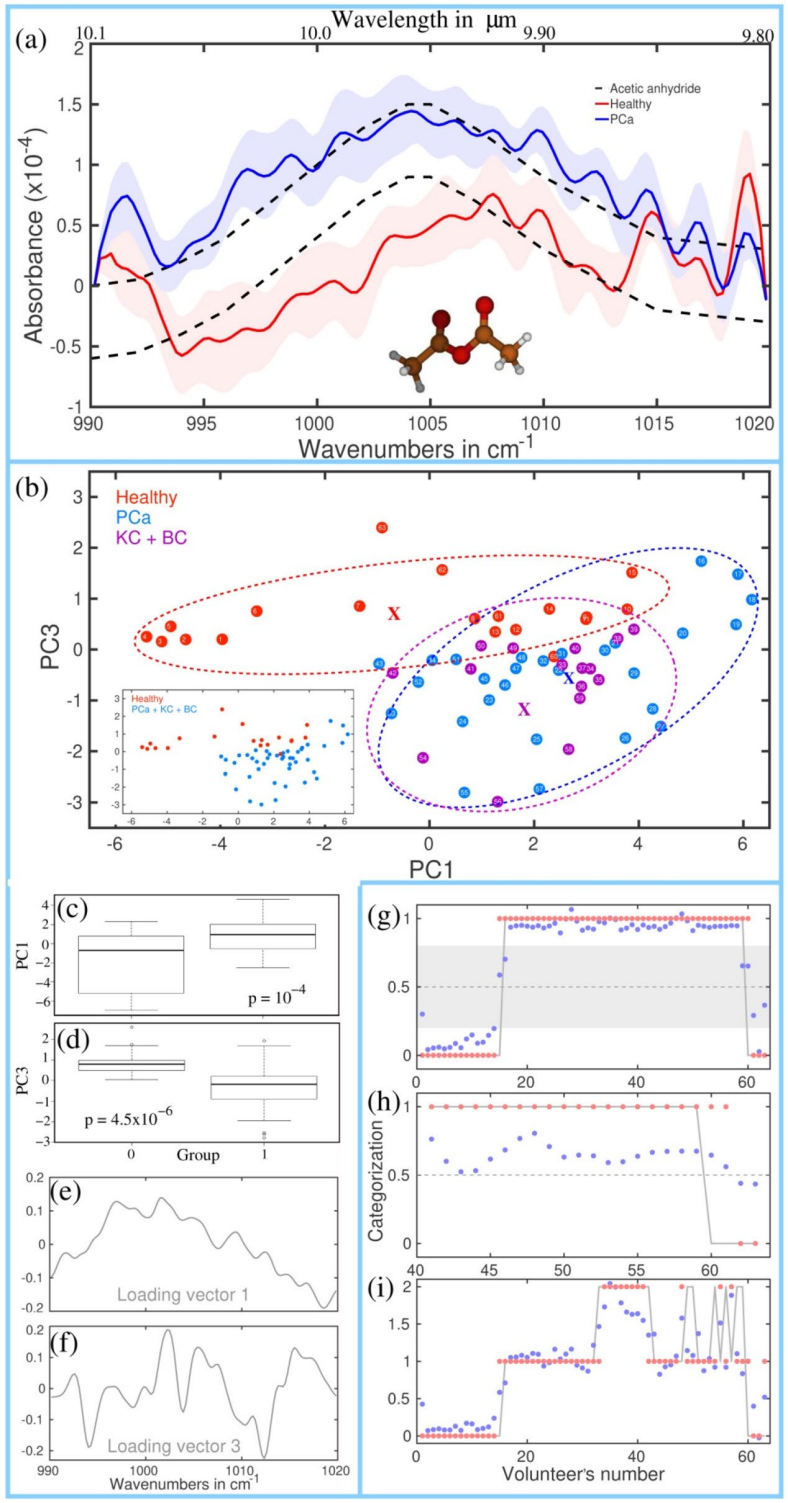
The SR centered at 1005 cm^−1^. (**a**) The average absorption spectra for healthy and PCa groups (other groups are shown in Supplementary Fig. S1). Oscillations visible for the range > 1010 cm^−1^ (especially at 1015, 1019 and 992 cm^−1^) are due to the contribution of CO_2_^21^. Bottom: 3D molecular structure of AA. The corresponding animation: (3D vibration of AA at 1005 cm^−1^.avi). White balls: hydrogen atoms, red—oxygen, brown—carbon. Shaded areas: deviations of the absorption in each group. Dashed lines: the absorption spectrum of AA taken from Ref.^21^. (**b**–**f**) Results of PCA, namely PC3 vs PC1 (**b**), plot boxes (**c**,**d**) and the corresponding loading vectors (**e**,**f**). Labels 0 and 1 in (**c**,**d**) represent healthy and PCa groups, respectively. (**b**) ellipses are shown for visualization, with the corresponding centers marked by “x”. The inlet is visualizes the separation between the healthy and cancer groups. (**g**–**i**) results of the supervised analyses. Category “0” corresponds to a healthy group, “1”—to a cancer group. (**g**) LOOCV analysis of the healthy and cancer (PCa + BC + KC) groups. Shaded gray zone 0.2–0.8 shows a number of points between the categories 0 and 1. (**h**) blind analysis taken for 4 healthy and 19 cancer patients (PCa + BC + KC). (**i**) 3-category analysis (sevenfold, 10 repetitions). Category “0”corresponds to the healthy group, “1”—to the PCa group and “2”—to the (BC + KC) group.

A comparison of the average spectra for cancer and healthy groups revealed 7 SRs (Table 1). Figure 1a illustrates one of these, the other SRs can be found in Supplementary Sect. 1 of the Supplementary Materials (SM). They were then statistically analyzed by unsupervised and supervised techniques. Further investigation of SRs in relation to volatile metabolites allowed us to hypothesize about processes leading to their variations in cancer as well as about their transportation in the body to the lungs (the “Discussion” section and Supplementary Sects. 6, 9 of SM). It has to be noted that one SR can represent several metabolites.

No correspondence was found regarding smoking habit, high methane concentration in breath, age, confounding current or previous diseases, high blood pressure or urine and stool biochemical content with the cancers that we analyzed. Results of PSA analyses of the patients under study were found ambiguous.

### Unsupervised analysis (PCA) + ANOVA

Five from seven SRs clearly differentiate the groups under study, i.e. demonstrate a p-value < 0.05 (Table 1). An illustration of analysis applied to the first SR is shown in Fig. 1b–f, others’ results are combined in columns 5, 6 in Table 1. We note that the first loading vector in (e) corresponds to the detected shape of SR. In contrast, the third loading vector (f) demonstrates spectral features visible on the shoulders of the experimental absorption spectrum at 992 cm^-1^ and at 1015–1020 cm^-1^ (presumably, due to CO_2_^21^). This validates the following supervised analysis of SRs in terms of the fitting metabolite curves that we performed (next section). Specifically, the highest absolute variances correspond to well-determined quasi-periodic spectra with high absorbance at 1050 and 2170 cm^−1^ (Supplementary Figs. S5–S6), for which the contribution of experimental noise is expected to be reduced. The variances related to PC1 are also high for 530 and 1005 cm^−1^ (Fig. 1 and Supplementary Fig. S2).

Analysis of the corresponding clouds of the data PC2 vs PC1, PC3 vs PC1, and PC3 vs PC2 visualized by ellipses (shown in Fig. 1b) revealed that healthy and PCa groups are comparable for all SRs in terms of the ellipse squares. The box plots demonstrate a similar ratio (Fig. 1c,d). The BC + KC cloud is more compact, “sitting” inside the PCa cloud. The reason for that can be limited statistics gathered for this group. The centers (marked by crosses) of these clouds, defined with the help of ellipses, are very close.

The enumeration of volunteers (Fig. 1b) allowed us to find that (i) the separation of a patient with a heavy cancer stage is maximal from the ellipse of a healthy group and (ii) the position of a light-stage patient is close to the healthy group. Specifically, BC patient #56 with the highest grouping score (see “Methods”), is located on the plot at the longest distance to the healthy group center. Patient #44 who underwent radiotherapy is close to the healthy group center. For further comparison with other analyses, we also noticed that patients #16 and #59 are close to the healthy cloud, and a healthy volunteer #60 is close to the cancer cloud. SR at 1005 cm^−1^ demonstrates the best separation of healthy and cancer groups, similar to the of supervised analysis results below. Possible reasons for that are presented in the “Discussion” section.

As we used two independent sets of samples (see “Methods” and a subsection below), we applied PCA + ANOVA to analyze bias between them. There are several reasons for bias (see the “Discussion” section) and its quantitative estimation is important from practical points of view. For example, it can hint at the validity of supervised analysis that exploits truly independent training and evaluation sets, compared with k-fold validation (and its extreme case, leave one out cross validation (LOOCV))**.** PCA analysis (Supplementary Table S1 in Supplementary Sect. 2 in SM) demonstrates small but observable bias between the sets.

### Supervised analysis

Three tasks were performed. The first task (A) included repeated k-fold validation by using the entire set of collected data. The aim was to see how two-category classification (healthy vs PCa + KC + BC and healthy vs PCa) and three-category classification (healthy vs PCa vs KC + BC) work. The second task (B) used evaluation (blind) analysis applied to the truly independent set of data to verify the validity of two models developed for the training set. This task included analyzing of continuous distribution of the volunteers’ data between the categories to see their variations for each SR and each group under study. The third task (C) aimed to identify correlation between biopsy and metabolite-based gradings.

#### Repeated k-fold validation (Table 1)

Four SRs demonstrated an accuracy exceeding 95%. They include the tasks healthy vs PCa (column 7) and healthy vs PCa + BC + KC (column 8). A similar level of accuracy was reached for 3 SRs in the task healthy vs BC + KC (column 9). A 3-category task aimed to differentiate healthy vs PCa vs KC + KC) resulted in a surprisingly high specificity of 80% for 4 SRs (an illustration in Fig. 1i, see also Supplementary Sect. 3, Supplementary Table S2 in SM). It means that a healthy person with an accuracy not less than 80% can be identified as having no PCa, BC and KC. Yet, the highest sensitivity of BC + KC vs healthy (sensitivity 2) reached only 42%. The 3-category task makes sense because of the difference in absorbance (i.e. concentrations) for the three groups under study (Table 1, column 4 and Fig. 1a and Supplementary Fig. S1 in Supplementary Sect. 1 of SM). The technique that we applied for this task, by definition implies low bias in comparison to the following evaluation analysis with two physically independent sets of data were used.

#### Analysis of truly blind data via SVM classifier (Table 2 and Fig. 1h)

**Table 2:** Results of two-category blind analysis (healthy vs cancer). Task 1: 15H, 17PCa as training set and 4H, 11PCa as blind set. Task 2: 15H, 17PCa, 8BC + KC as training set, 4H, 11PCa, 8BC + KC as blind set. H: healthy, PCa, KC and BC: see the text. *Sens* sensitivity, *Spec* specificity, *thr* threshold for the second model applied to the training set, *SD* standard deviation. SRs at 1190 and 1203 were analyzed together.

Wavenumber, cm^−1^	Threshold 0.5				Threshold optimized for training set			
	Task 1		Task 2		Task 1		Task 2	
	Sens	Spec	Sens	Spec	Sens (thr)	Spec (thr)	Sens (thr)	Spec (thr)
	1	2	3	4	5	6	7	8
1005	91	50	100	50	73 (0.63)	100 (0.63)	47 (0.65)	100 (0.65)
530	27	100	53	50	0 (0.65)	100 (0.65)	21 (0.7)	100 (0.7)
1050	73	100	96	50	73 (0.5)	100 (0.5)	58 (0.65)	100 (0.65)
1190 + 1203	91	0	100	0	91 (0.5)	0 (0.5)	100(0.4)	0 (0.4)
1130	27	100	79	25	73 (0.45)	50 (0.45)	95 (0.4)	0 (0.4)
1170	9	25	89	0	9 (0.5)	25 (0.5)	89 (0.5)	0 (0.5)
2170	27	75	68	75	27 (0.5)	75 (0.5)	68 (0.5)	75 (0.5)

The different models for the training sets were developed in this task to optimize either the sensitivity or specificity, with their further verifications via the corresponding evaluation sets. Specifically, in frame of the training set, we applied two models. They were devoted to two combinations of data: combination 1 included 15H (healthy), 17PCa as the training set, and 4H, 11PC as the evaluation set. Combination 2 included 15H, 17PCa, 8 BC + KC as the training set, and 4H, 11PCa, 8 BC + KC as the evaluation set (see Fig. 1h). We used the intuitive threshold equal to 0.5 between two categories 0 (healthy) and 1 (cancer) in the first model. They were then verified in the evaluation set (columns 1–4). This model led to high sensitivity (columns 1, 3) but relatively low specificity in the evaluation set (columns 2, 4). In the second model, we applied variable threshold that optimized the training set’s specificity (columns 5–8). The following evaluation set proved the model demonstrating significantly higher specificity (columns 6, 8). The resulting sensitivity and specificity in the evaluation sets exceeded 90% for 3 SRs, and reached 100% for 2 SRs in terms of sensitivity and for 3 SRs in terms of specificity. To note, the conventional diagnostic performance called area under the receiver operating characteristic curve (AUC-ROC) does not reflect the true situation, at least for 1005 cm^−1^ due to its high accuracy resulting in a Г-shaped curve.

The first reason why the two model-approach shows different results is related to the data variations between 0 and 1. For this analysis, the entire set of data (63) was taken for LOOCV analysis (illustrated in Supplementary Sect. 4, Supplementary Fig. S7 of SM). The merit of variations was the number of data points in the range 0.2–0.8 that we call gray zone (illustrated in Fig. 1f, see also Supplementary Sect. 4, 4a in SM). We found that (i) the cancer data variations in gray zone are significantly lower than those for the healthy and (ii) the number of variations correlate with the achieved accuracy (Table 2, columns 3, 8). Namely, the lowest number of the data points in the gray zone belongs to SR 1005 cm^−1^ that in the same time demonstrates the highest overall accuracy. The second reason is based on the experimental conditions: the unbalanced number of healthy in the training and evaluation sets, and different signal-to-noise ratio in the two sets. The sensitivity–specificity optimization implies that the data in columns 1 and 6, and 3 and 8 of Table 2 can’t be used simultaneously. In practice, a combination of the highest sensitivity and highest specificity in the same time is not needed. In other words, the choice of the focus groups in the study defines one or another accuracy parameter to be maximized. The resulting sensitivity and specificity in the evaluation test exceeded 90% for 3 SRs, and reached 100% for 2 SRs in terms of sensitivity and 3 SRs in terms of specificity.

#### Correlation of conventional and metabolite-based analysis

A high accuracy demonstrated in differentiating PCa (and a combination of PCa + BC + KC) from healthy in (A) and (B) allowed to pose another, more in-depth question whether there is correlation between the stage grouping (see “Methods”) based on biopsy analysis and the category analysis performed in B. To answer it, we zoomed the range around category 1 for SR@1005 cm^−1^, namely subzone 0.8–1.0, and divided it into two subzones, 0.8–0.9 (bottom subzone) and 0.9–1–0 (top subzone). For the correlation merit, we used conventional biopsy-based parameters T, N, Gl, G, R applied for the cancer characterization and combined in Table 3. Their variations were also divided into two classes, with high stage grouping that included Gl > 7, T > 2, N = 1 etc. in the first class and with lower scores in the second class. No parameter showing reasonable correlation between the top subzone and the first class (correlation 1), and the bottom subzone and the second class (correlation 2), was found.

**Table 3:** Information about volunteers.

Group	Numbers	Average age (max. deviations, + −)	PCa: Gleason score (number of patients); KC and BC: Grade (number of patients)	T (number of patients)
**Training group**				
Healthy	15		–	–
PCa	17	64.1 (+ 13.9–23.1)	6(3); 7(10); 8(1); 9(3)	2(11); 3(6)
KC	3	68.0 (+ 4.0–4.0)	1(2); 3(2)	1(3)
BC	5	68.8 (+ 13.2–13.8)	2(1); 3(2); 4(2)	1(2); 3(1); 4(2)
**Blind group, evaluation set**				
Healthy	4	57.8 (+ 7.2–8.8)	–	–
PCa	11	68.6 (+ 11.4–8.6)	6(2); 7(4); 8(3); 9(2)	1(1); 2(6); 3(3); 4(1)
KC	5	64.6 (+ 11.4–14.6)	1(2); 3(3)	1(4); 3(1)
BC	3	63.3 (+ 11.7–12.3)	2(1); 3(2)	0(1); 3(2)

Let us now compare the results of unsupervised and supervised analyses for the groups under study, and certain volunteers. First, in the plot PC3 vs PC1 the cancer (PCa + BC + KC) group defined via k-fold validation (category 1, Fig. 1g) is more compact than the healthy group (category 0, see also Supplementary Fig. S7). PCA represents groups in similar square (Fig. 1b). Bearing in mind that PCA and k-fold validation/LOOCV use different analyses, we therefore conclude that they are truly complementary. For example, PCA + ANOVA could bring information about several metabolites under one SR’ umbrella (like loading vectors in Fig. 1e,f). Second, we found a correlation of the proximity of healthy patients #9–10 and #13–15 to the cloud of PCa patients in both analyses. Large separation of the healthy data cloud #2–8 from the cancer category 1 (illustrated in Fig. 1f and Supplementary Fig. S7 in SM) correlates with the result of PCA (Fig. 1b). For the cancer cases, data of patients #16, 44, 59, 60 correlate with PCA results. Continuous (i.e., between the categories) distribution of the volunteer-related data, as can be seen (Fig. 1f, Supplementary Fig. S7 and Supplementary Table S3 in Supplementary Sect. 4a in SM), alters from one SR to another. These variations make the identified SRs complementary, thus enriching the knowledge about volunteers.

### Revealed metabolites

A panel of revealed metabolites that distinguished the healthy and cancer groups is summarized in Table 1 (column 2). The arguments supporting the metabolite identification and the literature demonstrating links between the revealed metabolites and cancer can be found in Supplementary Sect. 5 of SM.

## Discussion

In the following, we will rely on the validity of the identified metabolites extracted from the corresponding SRs.

Known papers represent focused studies on specific cancer, but not on a combination of cancers. The analyses presented here demonstrates that PCa, KC, BC have partially similar sensitive metabolites that differentiate them from healthy. If so, there must be a common source of the corresponding metabolites concentrated in the urogenital system. Inflammation-related carcinogenesis is one possible scenario among several alternatives. Applied to our study, it includes infection of the urogenital system by bacteria that can happen via several pathways. There is limited knowledge between the link of gastrointestinal microbiota and cancers of the urogenital system. Recently, it was shown that abundance of gut uropathogen increases the risk factor of bacteriuria and urinary tract infection, at least for some bacteria^22^. Several practical arguments are collected so far that support the inflammation-related bacterial carcinogenesis of the urogenital system (Supplementary Sect. 8 in SM). In this regard, similarities can be considered to the bacterium *Helicobacter pylori* leading to significant risk of gastric cancer (Supplementary Sect. 8 in SM).

Propionibacterium, namely *P. acnes* (*PA*) is one of the most promising candidates for the bacterial infection of urogenital system discussed in the literature^23^. We consider that under certain environment (for example, glucose that present in most of the cases in the urogenital system), the bacterium metabolism produces SCFAs as an energy source necessary for the tumor growth^24^, as well as AA and AD. As AA demonstrates the best performance in distinguishing the groups under study, let us discuss it in detail. Although AA can formally be considered as a derivative of acetic acid representing one of SCFAs, a realistic scenario of its production in the body is based on a chain of reactions in bacteria starting from AD (Supplementary Sect. 6 of SM). It has to be noted that AA is not an end product and its lifetime in the body is limited to minutes before the transformation to acetic acid via reaction with water, or to other products via acetylation (Supplementary Sect. 7 of SM). This makes its detection hardly possible in case of urine but still realistic via exhaled air that quickly extracts metabolites from the bloodstream (Supplementary Sect. 6 in SM). In acetylation, AA is the source of acetyl group necessary for complex protein modifications (an illustration in Supplementary Sect. 7 of SM). Specifically, acetylation alters local chromatin structure responsible for DNA packaging, and thus DNA replication and gene expression. These processes occur differently in healthy and cancer cases. Not only acetylation and deacetylation (Supplementary Sect. 7 of SM), but also methylation is another important molecular mechanism revealed in BC, with their prognostic and diagnostic markers^25^. Suppose the suggestion about initial bacterial infection of the urogenital carcinogenesis is correct. In this case, the ability of AA to distinguish (categorize) PCa and a group of KC + BC from healthy (Fig. 1f,h) can be explained by different bacterial contamination of kidney, bladder and prostate. It also offers a possibility of bacterial eradication via antibiotic therapy prior to carcinogenesis.

AA demonstrates the largest gap between 2 clouds of data (healthy and cancer) attributed to 2 categories (Fig. 1f,g and Supplementary Fig. S7 in Supplementary Sect. 4 in SM, and Tables 1, 2). The value of the gap can be considered another, more fine characteristics of the categorization quality (Supplementary Table S3 in Supplementary Sect. 4a in SM). We hypothesize that limited variations in each cloud and a significant gap between them (in gray zone 0.2–0.8) found for AA, could indicate the existing threshold between the healthy and cancer states. There is evidence pointing the imbalance between acetylation and deacetylation of histone or non-histone proteins (with the involvement of AA) as a driving force of cancer^26^. The imbalance could have a threshold character of an unstable situation that can lead to tumor initiation and progression towards category “1”, thus explaining the absence of participants’ data in gray zone. Other metabolites discovered in this study are involved in carcinogenesis mainly in a continuous (i.e. non-threshold) manner. They probably would help in detecting cancer at early stages via either their positioning between categories “0” and “1” or via using bio-passports^12^. The latter is based on “the islands of stability” approach described below.

It is noteworthy to stress here the endogenous nature of AA and AD. There is no link between AA, AD and medication: all the groups of patients took either different medication or no medication at all. Previously, AA and acetic acid were found as an environmental risk factor for PCa^27^.

Like AA, SCFAs are also involved in the fundamental reactions like acetylation of histones, chromatin and other proteins defining critical changes in cells^28,29^. As a bacterial candidate, *PA* is known to produce high amount of several SCFAs*.* The bacteria can also produce its transformation to the corresponding esters*. PA* strains studied in vitro, demonstrated SCFA-specific effect on acetylation/deacetylation^30^. SCFAs can also regulate cell functions by another way, namely through the activation of metabolite-sensing G-protein coupled receptors GPR41 and GPR43. It was demonstrated that PCa cells expressed GPR43 and GPR41^31,32^. It has to be noted that several types of cancers including PCa and BC were already associated with an increase in long fatty acid synthesis^33^.

As cancer is a heterogeneous, complex disease, one can expect a set of specific up- or down regulated metabolites. The panel of metabolites revealed in our study (Table 1) is somewhat limited but each of them demonstrates high sensitivity and specificity. It poses the question of whether only one of them can be used for reliable PCa diagnosis or diagnosis of cancers of the urogenital system. Obviously, the answer is not, if we want to realize both high sensitivity and high specificity^7^. There is an additional complication that makes the answer not trivial. How trustful the detection technique (tool) and the statistical analysis used in a study? Let us first comment on the complication related to the tool. The fact that GC–MS used in 99% of the corresponding metabolite-related studies during the last decades, has not yet demonstrated so far accuracies exceeding 95% in distinguishing healthy and diseased groups, provoking the question of why it is so? A partial answer can be found in the paragraph after the next one. As for the statistical analysis, we show that one type of analysis, namely conventional k-fold validation, demonstrates very high accuracies (Table 1). In contrast, the accuracy by analysis dealing with two truly independent data sets via SVM is significantly lower (Table 2). Which technique is more trustworthy? In our opinion, only a combination of reliable statistical techniques should be used in order to present trustful results of the study. For longitudinal studies, bias in data is obviously a problem. In our research, SVM analysis was applied to the second set of samples collected 1 year later than the first set. PCA analysis (Supplementary Table S1 in Supplementary Sect. 2 in SM), as well as SVM (Table 2) demonstrate some, but not significant, bias between them. This result is somewhat encouraging because (i) the spectrometer’s parameters changed during the period between the two sets of measurements and (ii) a breath sampling collection of the two sets was produced by other medical staff.

Metabolites collected in Table 1 demonstrate different sensitivity or specificity or error in distinguishing healthy vs PCa + KC + BC, or vs PCa or vs KC + BC. A related question that has to be posed in this discussion is whether the probability is high that a metabolite from the panel is altered naturally in the body of an individual, i.e. without any relation to cancer. Only a broader study can draw a reliable conclusion in this regard. In our opinion, the limits of natural deviations of specific metabolites should be analyzed along the way we recently demonstrated using “the islands of stability” approach^12^. It uses a multidimensional metabolite space (each metabolite represents a dimension) developed to visualize limits of natural variations of individuals’ metabolites. More generally, the approach could allow to (i) detect the onset of a disease by monitoring the position of “the island” of an individual and (ii) distinguish the individuals (i.e., defining a truly individual bio-passport). Similarly, we can build a multidimensional metabolite space of diseases. This space presumably has overlapping dimensions (i.e. metabolites) for different diseases, but the entire set of dimensions and the position of a certain disease in it (i.e., its unique island) is expected to be special. As an illustration, propionates that we revealed for cerebral palsy^20^ and cancers of the urogenital system in this study, could serve as an example of such an overlap in one dimension.

Let us now comment that AA was not identified previously in biofluids of patients by means of mass-spectrometry methods, the most convenient technique for analysis of metabolites in gas and liquid phases. First, there are ten molecules with the same formula C_4_H_6_O_3_ (molecular mass 102.09 amu). These molecules are hardly distinguishable by GC–MS but are well distinguishable by mid-infrared spectroscopy that we applied in the fingerprint region. Second, in addition to those ten, there are several molecules with the difference in molecular mass less than 0.1%, thus making the identification even more difficult. Because of that, metabolites identified by GC–MS in most of research papers, mainly contain heavy molecules for which the identification ambiguity (“molecular mass degeneracy”) is less. In principle, a contribution of GC should help in identification of isomers in a mixture, but we are not aware of any published results devoted to distinguishing molecules under the umbrella of the C_4_H_6_O_3_ formula. In this sense, mid-infrared spectroscopy and GC–MS are complementary, with the border between them for molecular mass around 120 amu. The first technique is more appropriate for light volatile metabolites whereas the second one—for heavy ones in gaseous and liquid states.

The hypothesized transportation scheme based on the validity of the revealed metabolites and presented in Supplementary Sect. 9 of SM can explain the elevated level of metabolites in breath (Table 1). For up-regulated metabolites (column 4 in Table 1), it includes an additional channel connecting the urogenital system and lungs via the bloodstream. Another scheme should explain the down-regulated carbon dioxide based on the Warburg effect, and ethyl pyruvate.

A more advanced scheme for up-regulated metabolites should establish quantitative relations between the concentrations of the identified metabolites. For example, the gut microbiota produces acetic, butyric, propionic SCFAs in a ratio of 60/20/20^34^. One could expect a similar ratio in our case because (i) the main derivatives of SCFAs are present in channel 1 of the hypothesized transportation scheme (Supplementary Fig. S9 in Supplementary Sect. 9 of SM) and (ii) if carcinogenesis of the urogenital system is initiated by bacterial infection, for example *PA,* it may also provide a similar ratio. We are not aware of any study regarding the SCFAs ratio of the *PA* metabolism in the urogenital system. Nonetheless an in-vitro experiment demonstrated various ratios for different strains of *PA*^24^. Data collected in Table 1 (column 4) together with two assumptions: (i) similar esterification of SCFAs and transportation of esters to the lungs, (ii) a ratio of AD/acetic acid equal to 0.5 (Supplementary Sect. 6 in SM), led to the SCFAs ratio 70/15/15 that supports a dominance of the channel 1, namely the gut-lungs axis in the hypothesized transportation scheme. The proposed transportation scheme calls for the expected relative value of the metabolite variations between the groups equal to the ratio of the two channels.

Showing > 95% of sensitivity and specificity, we have a solid argument for early cancer detection. An additional argument supporting this hope includes the patient without the primary tumor (i.e. T0; after resection made in frame of biopsy, with further surgery operation) who was identified as having cancer. To demonstrate an early (pre-symptomatic) cancer detection, the tool we used here must be modified. Advanced laser-based spectroscopy based on powerful broadband mid-infrared generation^18,19^ together with more aggressive physical and digital suppression of water, carbon dioxide, methane (and other molecules present in breath with high absorption^35^), should be performed in concert in order to push the part-per-billion barrier in the entire range 500–4000 cm^−1^. For practical use in the foreseeable future, a laser spectrometer based on a set of quantum cascade lasers operating at the wavelengths of identified biomarkers, can be considered. These lasers are compact, demonstrating Watt-level radiation in mid-infrared and therefore can be combined with a compact but long multipass gas cell. All the steps, being progressed, could allow for detecting an early stage of PCa and other cancers.

Similar to Ref.^35^, the analysis of the experimental data that we performed revealed low correspondence of the metabolite-based and conventional biopsy-based gradings.

Summarizing, we conclude that the small study results presented here, outperform the results of conventional PSA diagnostics and recent urine-based results exploiting volatile metabolites^7^. We demonstrate for the first time a diagnostic approach differentiating not only PCa vs healthy, but also healthy vs KC + BC, with an accuracy exceeding 95%. These results fully rely on the analysis of the spectral ranges that differentiate the groups under study. If the corresponding metabolites are identified correctly, the following outcome could be formulated. The analysis of three types of cancer revealed one more argument supporting the bacterial-initiated carcinogenesis scenario of the urogenital system. Namely, AA, AD and derivatives of SCFAs elevated in breath of the urogenital cancer patients, are the products of the bacterial metabolism in the urogenital system. Spectroscopy of breath holds the promise for the cancer detection at early (pre-symptomatic) stages.

## Methods

### The spectroscopic and breath collection techniques

The spectrometer and its performance for real breath samples were described in Refs.^12,35^, and we therefore present only its key parameters here. We used a Fourier spectrometer Bruker Vertex 70 operating in the spectral range from 500 to 4000 cm^−1^ in conjunction with a 4 m 2 L “White cell” (Bruker) that contains the breath for the measurement, and with a liquid nitrogen cooled MCT detector. At the highest achievable sensitivity of the detector (necessary for detecting spectral features that show statistically different absorbance for the groups under study), the baseline demonstrated nonlinear distortion (Supplementary Fig. S10 in Supplementary Sect. 10 of SM) corrected by home-made software written in Matlab. For all measurements, 0.5 cm^−1^ spectral resolution was used. The minimum detectable concentration in a range of 1000 cm^−1^ (having low influence of residual water) achieved 50 ppb (parts-per-billion, Supplementary Sect. 11 of SM). The range > 1100 cm^−1^ is more affected by water and, in combination with the spectral sensitivity of the detector, demonstrates a smaller signal-to-noise ratio. A breath sample comes from a Tedlar bag (Sigma-Aldrich) into the cell via a gas system containing a water condenser that suppresses the amount of water by a factor of 2500. The accuracy of the measurement system defined by repetitive measurements of a known sample is + − 2.5%. All measurements were taken in one clean room at room temperature and humidity between 40 and 60%. Single-use Tedlar bags were used for both groups of individuals under study. To verify that SRs do not originate from the surface of Tedler bags, additional spectral measurements with direct breathing into the gas system were made. Sample collections in the bags had been made in two clean rooms, each of them was used for both healthy and cancer volunteers. In order to check the influence of conditions in the rooms on the resulting absorption spectra, we collected breath of a healthy person in both rooms. In the spectral range of our main interest, we did not find any noticeable difference. We found that the breathing procedure is not of high importance, in spite of wide discussion in the community^12^. We relied on normal breathing available for all the patients who participated in this study.

Post-processing of the experimental spectra was based on several steps described previously^12,20,35^. The first step included the baseline correction of the experimental absorption spectrum in the range 500–4000 cm^−1^. For the flattening necessary for further analysis, the spectrum was split in spectral ranges of approximately 100 cm^−1^ width. The second step included a Gaussian filter applied to the raw data in order to smoothen the spectral noise. As a priori it is difficult to estimate the appropriate filter width, we applied a filter of variable width during the comparison procedure until the classification accuracy was maximized. All spectral points were mean-centered (the mean was subtracted) and normalized by dividing through their standard deviation.

### The molecular identification

First, it should be stated that the molecular identification is a high probability guess based on the fitting procedure, and so far, a standard operating procedure for that does not exist. During the initial step of the fitting procedure, we checked a coincidence of the maxima in the experimental spectral features (for illustration, see Fig. 1a) with those of candidates taken from the compendium^36^ by using free NIST Chemistry WebBook^37^. The degree of fitting was checked qualitatively via visual inspection and quantitatively by using the least mean square fitting algorithm. Special care was taken to the absence of additional spectral peaks of the molecular candidate not revealed in the experimental spectra. Spectral analysis of pure molecules led to the conclusion that any peak asymmetry at the scale 10–30 cm^−1^ in a real breath sample could be explained by the contribution of another molecule. The compendium^36^ contains more than 800 volatile metabolites detected by mass spectrometry, whereas the NIST database contains at least hundreds of mid-infrared spectra of organic molecules. Unfortunately, the NIST spectra are not available in digital form, preventing the comparison with a spectral resolution better than 5 cm^−1^. Other available databases^21,38^ provide more precise data but the number of molecules is limited. A discrepancy below 50% in width between the measured spectral peak (both, a pure substance and the breath sample) and the extracted from the databases were considered as acceptable.

The next step of the identification contained two comparisons. For the first comparison, several best candidates chosen from the initial step were purchased (Aldrich) and measured by using our spectrometer with the same resolution and other conditions that we used for detecting breath samples. In few cases, the candidates were toxic and therefore only their known spectra were used.

For the second comparison, another free spectral database^38^ was used, where in addition to the printed spectra (in analog form), the positions of the main absorption maxima were presented in digital form.

### Statistical analysis

We applied two types of statistical analysis: unsupervised, namely PCA + ANOVA and supervised that included LOOCV, k-fold cross validation and blind evaluation of the independent set of data (see the next section). Mathematica was used for data preparation (smoothing and normalization) and SVM was run in R. SVM was chosen because it demonstrated the best classification scores among other approaches in our previous studies. Both types of analysis allowed for monitoring individuals of each group under study. Each individual was represented by a sample number. This option offered a way of including confounding factors into analysis, either via applying rigid categorization or, mainly, via using gray zones between the categories. Categories were defined in the following way. In the case of two-category analysis, category “0” was attributed to healthy, “1”—to PCa or to (PCa + BC + KC). In the case of three-category analysis, category “0” was attributed to healthy, “1”—to PCa and “2”—to (BC + KC). The healthy group was considered as the main reference group. The two- and three categorization tasks were applied to the entire set of data (63 individuals) by using LOOCV. The two-categorization task included analysis of PCa vs healthy and cancer (PCa + BC + KC) vs healthy, represented for each individual and for each SR. Three-categorization task included analysis of BC + KC vs PCa vs healthy, with two sensitivities and one specificity. In this case, accuracy was defined as the ratio of correctly identified samples of the task to all samples. Sensitivity 1 was defined as the ratio of correctly identified PCa in the task to all PCa. Sensitivity 2 was defined as the ratio of correctly identified (KC + BC) in the task to all (KC + BC). Specificity was defined as the ratio of correctly identified healthy in the task to all healthy.

Monitoring of the continuous distribution of individuals’ data (0.2–0.8 defined as gray zone) between rigid categorizations (0 and 1) gives a possibility to analyze (1) individuals more accurately in terms of their state and (2) compare different SRs (or the corresponding metabolites) in terms of the data population in gray zone. To do this, LOOCV-based categorization should be applied to the entire set of data. A merit function for the best SR (i.e. the best accuracy in distinguishing the groups) implies the minimal amount of points in gray zone.

The evaluation (blind) analysis was performed with the help of SVM applied to the data collected and processed 1 year later than the training set (Table 3). Two types of models were developed for the training set: first, with the categorization border 0.5 (healthy is 0, cancer is 1) and second, with the flexible categorization border in a way that maximizes either the sensitivity or the specificity. The corresponding code was written in Mathematica. This model gives a possibility to maximize either sensitivity at certain specificity or other way around. The model makes a practical sense because can be applied to the data analysis of a certain individual.

### Information about samples, sampling procedure and safety measures

The breath sample collection was done in a hospital room having fresh air, after the breakfast. After the collection, the samples within two days were moved to another room for spectroscopic measurement. Storing of the samples was arranged at 4 °C. The measurement procedure is explained in detail in Ref.^12^. The second set of samples has been collected 1 year later, during the COVID-19 period and therefore, two precautions were performed. First, all patients in the hospital passed the COVID-19 PCR test with negative result prior the sampling and second, the entire pathway of a breath sample was controlled from the Tedlar bag to the exit from the measurement cell, in order to provide extra safety conditions for the researchers. This set was collected by another medical staff instructed by the previous one. In addition, a video teaching course for the sample collection was used. Because of technical reasons, this set was measured with lower water suppression (factor 2200 instead of 2500 for the first set) and therefore, larger noise or bias in data was expected.

### Subjects of the study

The participants of this study were informed by video and word instruction about the breathing procedure and gave then their written consent prior the breath collection. Breath of the PCa, BC and KC group was analyzed after the diagnosis and just before the surgery. Surgery was performed as prostatectomy for PCa, radical cystectomy with urinary diversion for BC and total/partial nephrectomy for KC. Patients with PCa and BC were diagnosed based on histology analysis (biopsy/transurethral resection) prior the surgery. Patients with KC were diagnosed with either CT scan, MRT or contrast-guided ultrasound. Data about participants of both sets are summarized in Table 3. The training set included 5 smokers and 2 patients with metastases. The evaluation set included 3 smokers and 1 patient with metastasis. One patient with BC had a T0 tumor in the final histology after cystectomy. This was the result of a thorough previous transurethral resection. Another patient underwent radiotherapy before the breath collection. The following general set of parameters was used for the analysis: tumor size T, lymph nodes N, distant metastasis M, histopathological grading (Gleason score for PCa Gl, and grading G for BC and KC), stage grouping or clinical staging (a combination of T, N, M). The results of conventional urine and stool analyses were also used for the comparison of groups under study.

### Guidelines/regulations and ethical documents

The study was performed in accordance with the Declaration of Helsinki and relevant guidelines/regulations published by the European Medicines Agency's Committee. The necessary ethical documents and experimental protocols were issued and approved by the Ethics Committee of Ludwig Maximilian University of Munich. Reference Number: 19-190.

## Supplementary Information

Supplementary Information.

Supplementary Video 1.

## Abbreviations

BC: Bladder cancer
CO_2_: Carbon dioxide
GC–MS: Gas chromatography mass spectrometry
AA: Acetic anhydride
AD: Acetaldehyde
KC: Kidney cancer
LOOCV: Leave one out cross validation
PA: *Propionibacterium acnes*
PC: Principal component in principal component analysis
PCa: Prostate cancer
PCA: Principal component analysis
SCFA: Short-chain fatty acids
SR: Spectral range
SVM: Support vector machine
