# Supplementary material for: Towards reliable diagnostics of prostate cancer via breath
Source: Sci Rep. 2021 Sep 15;11:18381. doi: 10.1038/s41598-021-96845-z (PMC8443711; doi:10.1038/s41598-021-96845-z)
Supplement: Supplementary file 1 — Supplementary Information. [file 41598_2021_96845_MOESM1_ESM.docx]

**Supplementary Materials**

**1. Average spectra for different SRs.**

**
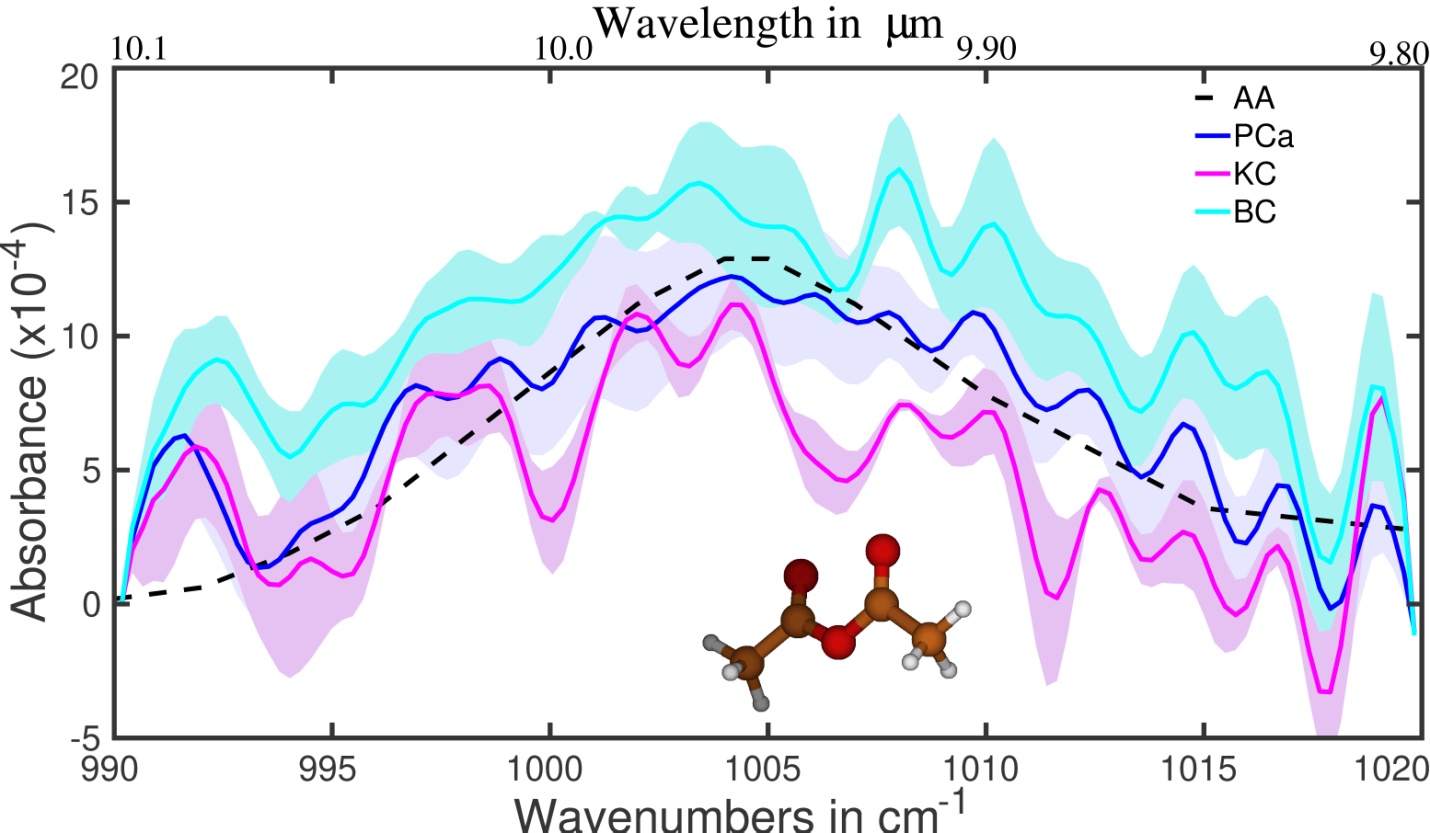
**

*Figure S1. Average absorption spectra at 1005 cm^-1^ for different cancer groups. Shaded areas: variations of the data within each cancer group. Dashed line: absorption spectrum of AA.*

**
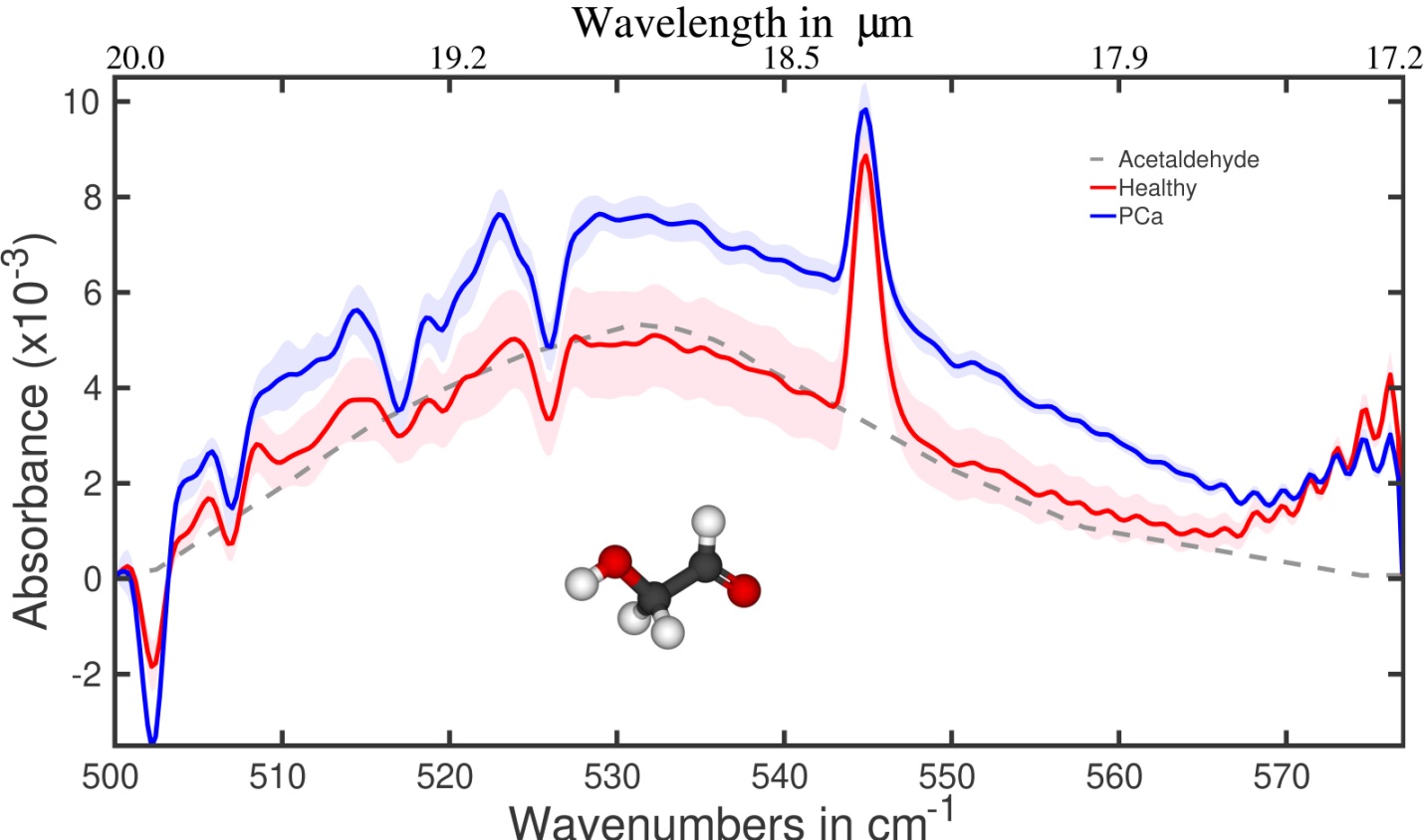
**

*Figure S2. Average absorption spectra at 530 cm^-1^ for different groups. Shaded areas: variations of the data within each group. Dashed line: absorption spectrum of acetaldehyde. The strong peak at 546 cm^-1^ belongs to CO_2_.*

**
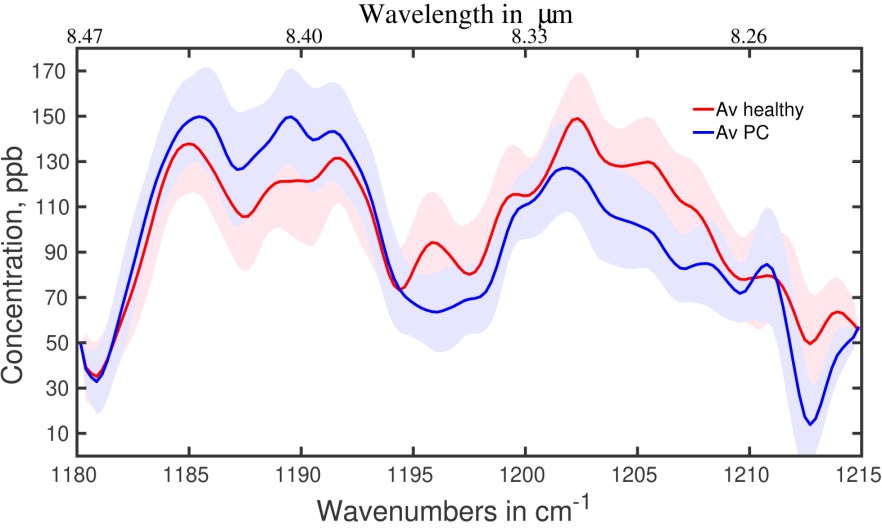
**

Figure S3a. The *SR that combines two structures.* A*verage absorption spectra at 1190 cm^-1^ (details see in Figure S3b) and 1203 cm^-1^ (details in Figure S3c) for the healthy and PCa groups. Shaded areas: variations of the data within each group.*

**
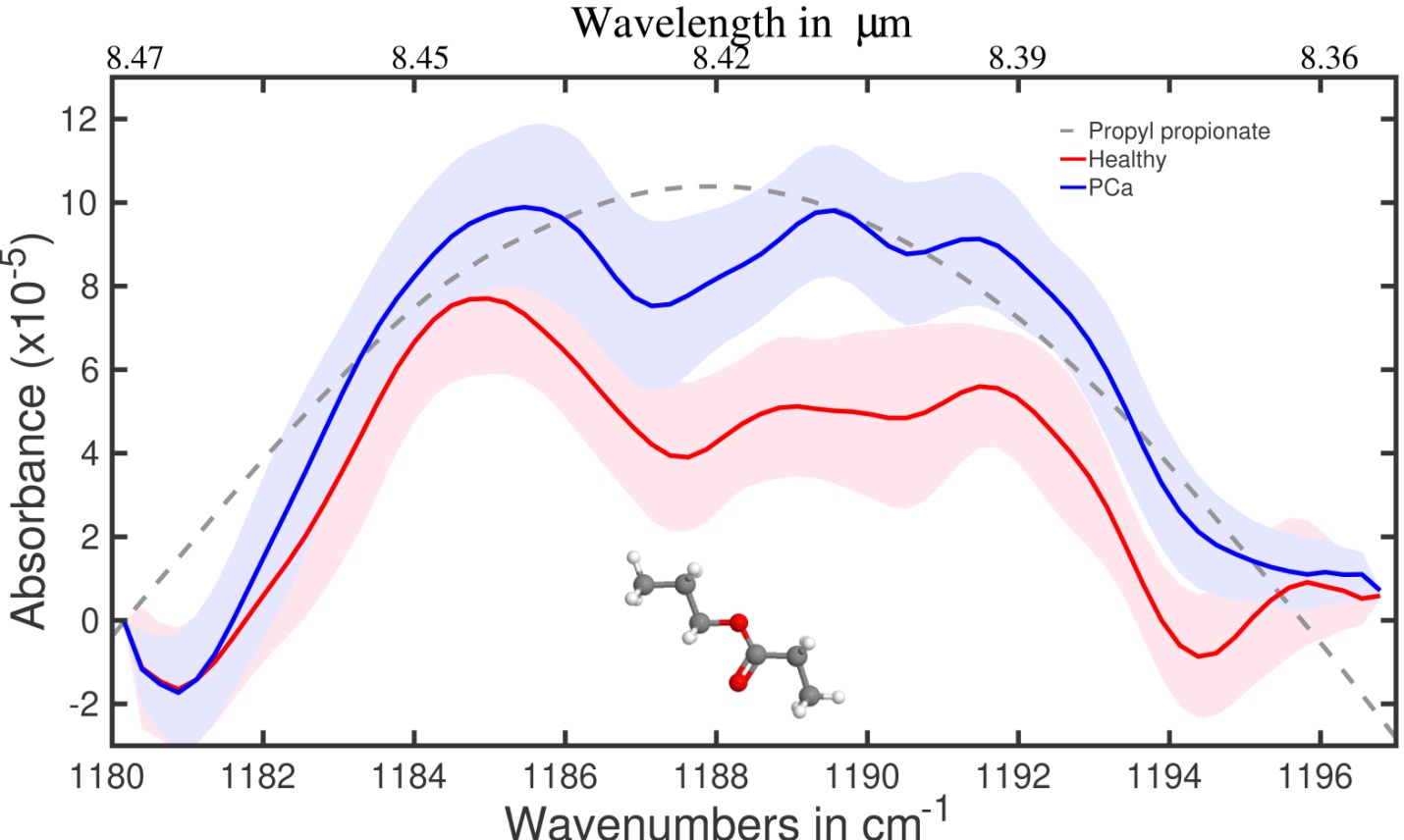
**

*Figure S3b. Average absorption spectra centered at 1190 cm^-1^. Dashed line: absorption spectrum of propyl propionate.*

***
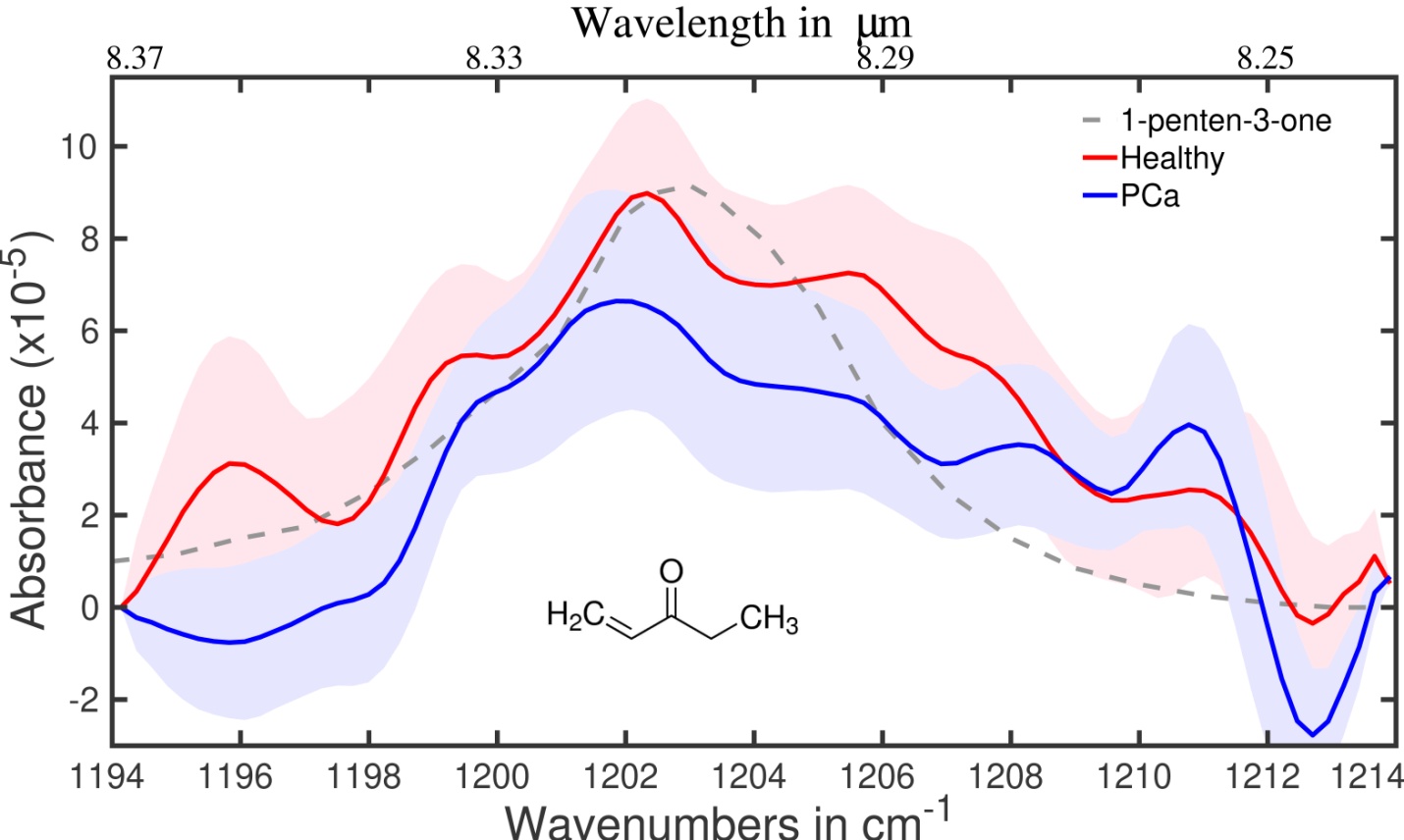
***

*Figure S3c. Average absorption spectra centered at 1203 cm^-1^. Dashed line: absorption spectrum of ethyl vinyl ketone (1-penten-3-one).*

**
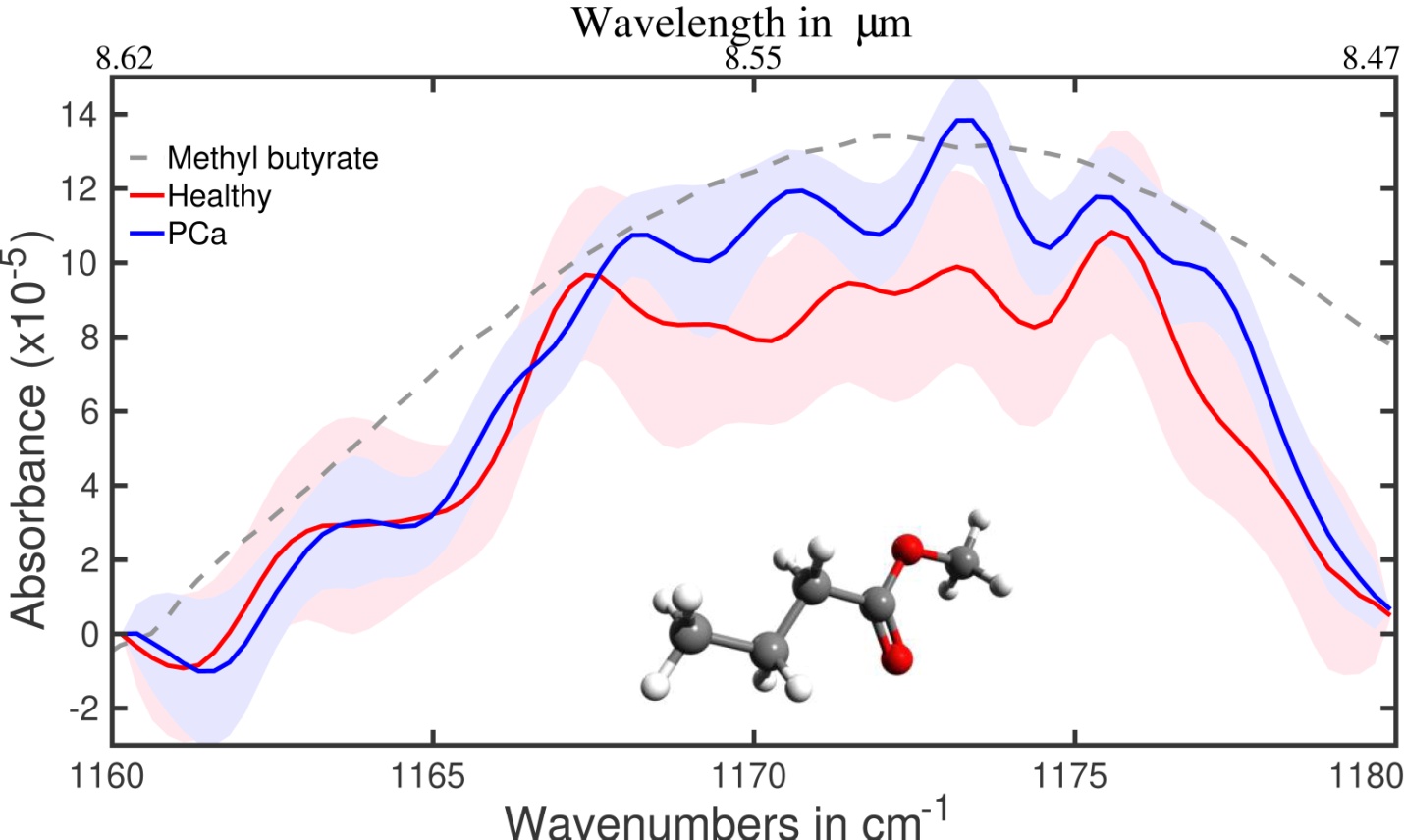
**

*Figure S4. Average absorption spectra centered at 1170 cm^-1^ for different groups. Shaded areas of different colors: variations of the data within each group. Dashed line: absorption spectrum of methyl butyrate.*

**
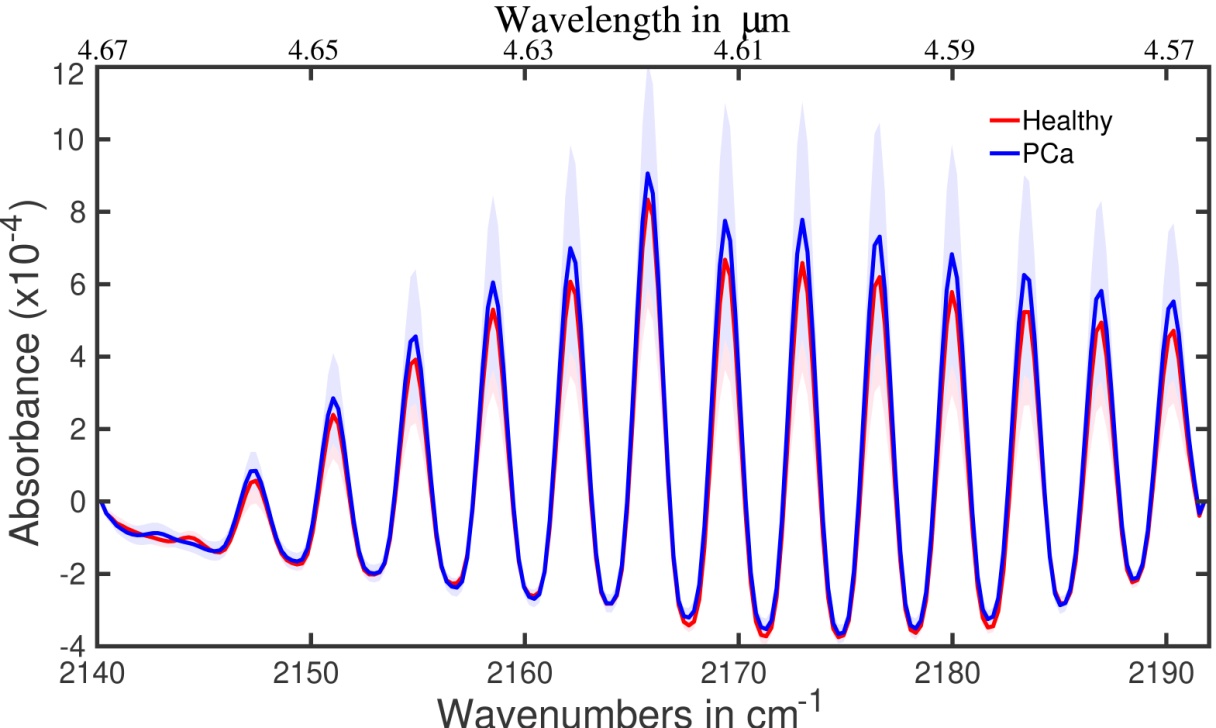
**

*Figure S5. Average absorption spectra centered at 2170 cm^-1^ and identified as carbon monoxide. The baseline was not corrected.*

**
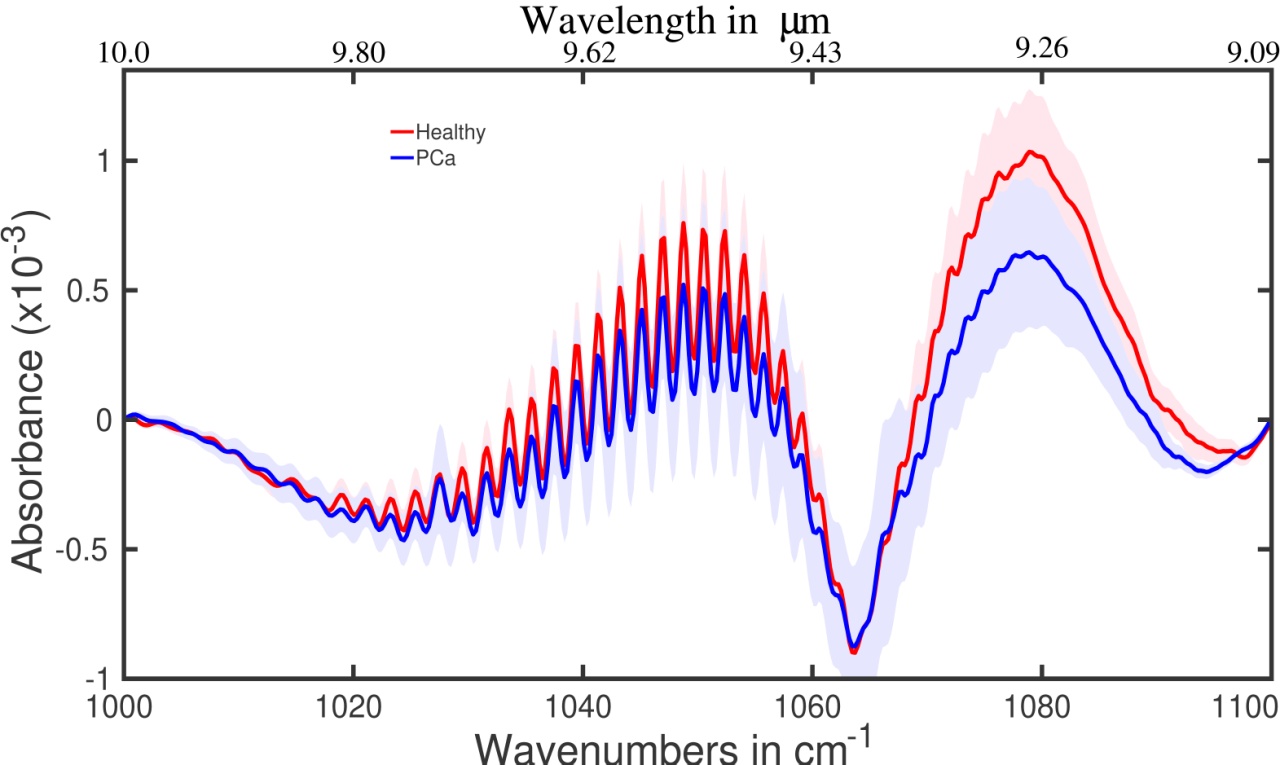
**

*Figure S6. Average absorption spectra centered at 1050 cm^-1^ and identified as carbon dioxide.*

**2. Bias between the two sets of samples.**


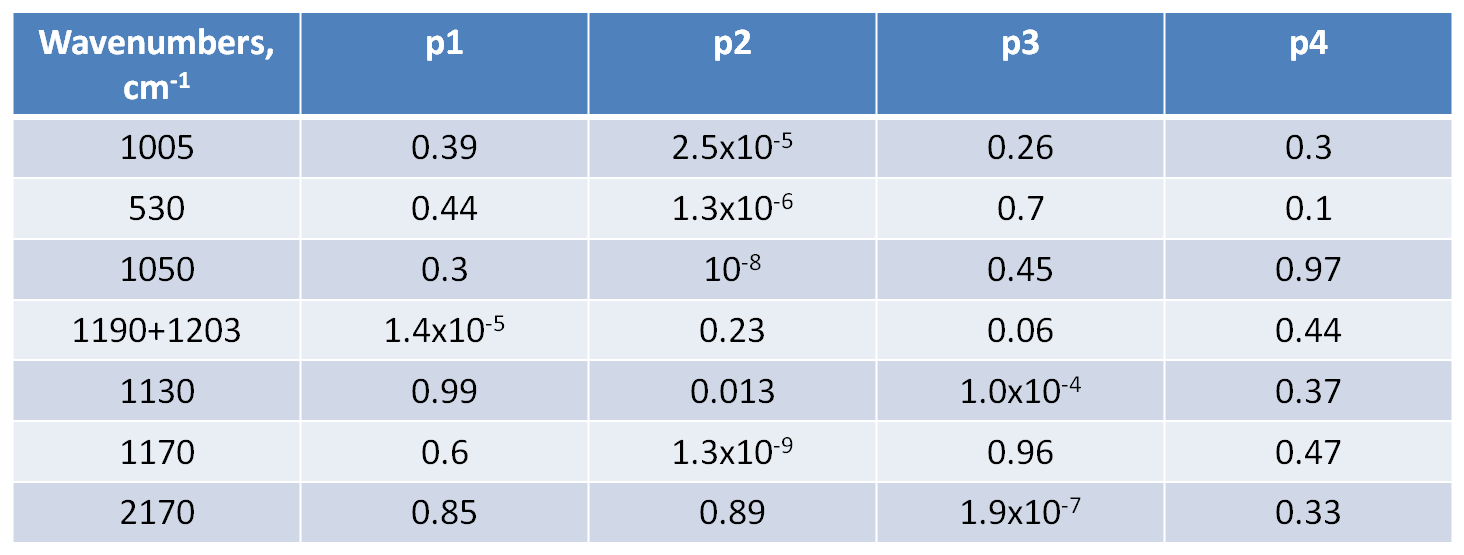


Table S1. p-values for calculating the bias at different SRs.

PCA+ANOVA was used to analyze how independent are the data of the first and second set of samples. A figure of merit was the p-value. In case of p>0.05, the data of the corresponding samples are dependant, and bias is expected to be low. For each of SRs, the PC1, 2 3, 4 were analyzed, with the outcome in the corresponding p-values. As one can see, each SR contains only one from four p-value <0.05. We conclude that bias is not zero, but small.

Bias was also estimated by using the results of SVM presented in Table 2. As one can see in column 8 of the blind set, SR 1130 cm^-1^ demonstrates significant difference with the training set. Variations of bias for different SRs can relate to different natural or induced by disease variations of the corresponding metabolite. As an example, methane is well known as a metabolite of large concentration variations [25].

**3. Table S2. 3-category analysis (7-fold, 10 repetitions). A description of accuracy, sensitivities and specificity can be found in Methods. In brackets: error, SD.**

| Wavenumber,  cm^-1^ | Accuracy | Sensitivity 1 | Sensitivity 2 | Specificity |
| --- | --- | --- | --- | --- |
| 1005 | 0.77 (0.02) | 0.92 (0.02) | 0.41 (0.07) | 0.84 (0.04) |
| 530 | 0.68 (0.03) | 0.98 (0.02) | 0.24 (0.08) | 0.61 (0.09) |
| 1050 | 0.67 (0.03) | 0.95 (0.03) | 0.06 (0.05) | 0.76 (0.09) |
| 1190 and (1190+1204) | 0.78 (0.03) | 0.95 (0.03) | 0.39 (0.08) | 0.85 (0.09) |
| 1130 | 0.75 (0.02) | 0.94 (0.04) | 0.34 (0.09) | 0.80 (0.04) |
| 1170 | 0.80 (0.03) | 0.96 (0.02) | 0.42 (0.07) | 0.89 (0.07) |
| 2170 | 0.63 (0.02) | 0.99 (0.01) | 0.18 (0.05) | 0.46 (0.05) |

**4. LOOCV analysis for different SRs.**

**
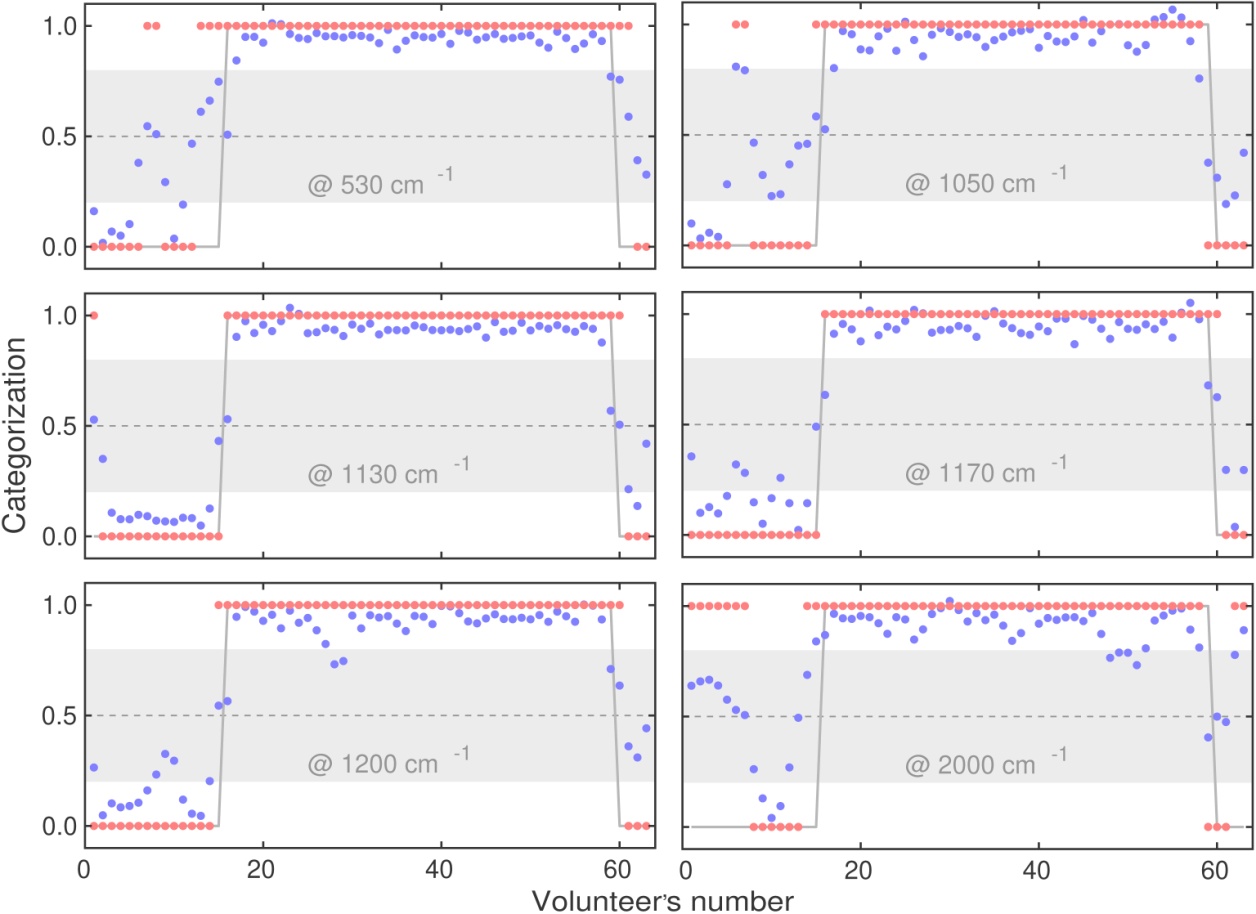
**

*Figure S7. A panel of two-category data classification via LOOCV for different SRs. Category 0 corresponds to the healthy group, category 1 - to the cancer (PCa+BC+KC) group. The SR marked as “1200 cm^-1^“, contained two SRs at 1190 and 1203 cm*^-1^*.*

**4a. Table S3. Gray zones: continuous distribution of the data between the categories.**

Category 0 corresponds to the healthy group, category 1 - to the cancer group. Gray zone was defined as 0.2-0.8; reference to Figure S7.

| Wavenumber, cm^-1^ | Healthy group, individual’s number | Cancer group, patient’s number |
| --- | --- | --- |
| 1005 | 1/15/60-61/63 | 16/59 |
| 530 | 1/6/ 7-9/11-15 | 16/17/59 |
| 1050 | 5-15/60-63 | 16/58/59 |
| 1190+1203 | 1/8-10/15/60/62/63 | 16/27/28/59 |
| 1130 | 1/2/13-15/60-63 | 16-18/15/59 |
| 1170 | 1/2/6-8/11-15/60/61/63 | 16/59 |
| 2170 | 1-8/12-15/60-62 | 16/48-52/58/59 |

The number of data in the gray zone is minimal for SR 1005 cm^-1^, thus showing the best categorization.

**5. Revealed metabolites**

As the revealed metabolites except carbon monoxide and carbon dioxide were not reported in papers of other research groups devoted to spectroscopy of biofluids, the results below i) should be considered cautiously and ii) need to be independently verified.

AA (CAS 108-24-7).

Figure 1a shows higher absorbance (proportional to the concentrations of the corresponding volatile metabolite) of SR centered at 1005 cm^-1^ in case of PCa and BC and KC (Figure S1 in SM) in comparison to the healthy group. Among those three, the absorbance of BC is the highest and the absorbance of KC – the lowest, is more elevated than that of the healthy group. Using the identification steps described in the Methods section, we concluded that this spectral feature could be attributed to AA. It was chosen as the best fitting candidate because of 2 reasons: firstly, the corresponding peak labeled in the Chemical Book [1] and [2] matches both the maximum (within 4 cm^-1^ for both groups) and the width of the curve. The asymmetry of the peak for the healthy and BC groups can be explained by the contribution of an additional metabolite centered at 1007 cm^-1^, absent for PCa and KC (Figure S1 in SM). Extra noise from water and large variations of carbon dioxide between the volunteers resulted in lowering the signal-to-noise ratio in the range 1120-1126 cm^-1^ of another characteristic AA peak [1]. Though it was observed for 60% volunteers, the averaging over the groups under study washed the peak out. To note, experimental measurement of the AA absorption curve has technical and safety problems. Secondly, it is present in Human Metabolome Database (HMDB) [3] and in the compendium [4].

The identified vibration of AA corresponding to the peak at 1005 cm^-1^ is shown in section 5 in SM. As it looks rather complex and specific, we want to stress the great difference between the identification of metabolites in gas phase and the identification of C-H, C=O (and other bonds) averaged over all the ensemble of the contributing molecules, available for analysis of liquid phase biofluids.

The reason why AA was never mentioned in literature as a disease-related metabolite looks obvious: AA is not stable and becomes transformed into acetic acid in biofluids containing water. It makes its detection hardly possible via conventional detection techniques. This metabolite will be discussed in detail in the Discussion section, emphasizing i) its production in the urogenital system and transportation to the lungs, and ii) possible links to carcinogenesis.

AA is an indirect product of bacterial metabolism generated after several biochemical steps (section 6 in SM). Notably, AA is deeply involved in carcinogenesis (section 7 in SM). This circumstance can explain its high accuracy demonstrated for the unsupervised (in terms of p-value) and supervised (in terms of sensitivity and specificity) analyses.

Acetaldehyde (AD; CAS 75-07-0).

Because its direct spectroscopic measurement is problematic (a decision of the International Agency for Research on Cancer in 2009), the Identification has been made using two databases [1, 5]. All aldehydes have strong overlapping absorption spectra in the spectral range 1700-1800 cm^-1^. Therefore, it makes difficult to reveal AD in this range [6,7]. In contrast, the range 500-560 cm^-1^ makes its unambiguous identification possible (Figure S2 in SM). AD is an oxygen-containing molecule known to be generated during specific alcohol metabolism pathways and can lead to the damage of the DNA, thus inducing tumor development. But the main channel of the AD production is bacterial metabolism [8], for example by Propionibacterium *P.acne* (PA) [9]. AD was found in kidney, urine, blood [3] and described in the compendium [4] for all biofluids in gas phase. Its relation to PCa was already identified [10].

Methyl propionate (propanoic/propionic acid, methyl ester; CAS 554-12-1).

A short-wavenumber part centered at 1190 cm^-1^ of a complex absorption structure (Figure S3 in SM) was identified as methyl propionate after its direct spectroscopic measurement and comparison with the experimental absorption spectrum. The metabolite is a derivative of SCFA, namely propionic acid. Like AA and AD, this metabolite is also related to the bacterial metabolism and can be found in the compendium [4] and in the HMDB database [3], though without any relation to oncology.

Ethyl vinyl ketone (1-penten_3-one; CAS 1629-58-9).

Because of the proximity of the SR centered at 1203 cm^-1^ (Figure S3c in SM) to the SR at 1190 cm^-1^ (Figures S3b), its statistical analysis was performed for the entire structure shown in Figures S3a. The SR was identified as ethyl vinyl ketone after comparing its absorption spectrum with the database [1] (matching of absorption peaks within 1 cm^-1^). The asymmetry pronounced at 1208 cm^-1^ is caused by another metabolite. An additional peak at 1122 cm^-1^, like in the AA case (see above) was not observed, with the same explanation. Ethyl vinyl ketone can be considered as an oxygenated hydrocarbon lipid molecule present in the HMDB database [3] and in the compendium [4], in both cases without direct links to cancer. Like CO_2_ in cancer (below), ethyl vinyl ketone linked both to glucose and acetyl-CoA is also down-regulated.

Methyl butyrate (butyric acid, methyl ester; CAS 623-42-7).

An SR centered at 1170 cm^-1^ (Figure S4 in SM) was identified as methyl butyrate after its direct spectroscopic measurement and comparison with the experimental absorption spectrum. The metabolite represents a derivative of SCFA. Similar to previous metabolites, it is a product of bacterial metabolism and can be found in [3], with relation to gastrointestinal cancer, but not to the urogenital cancer. The compendium [4] contains only butyric acid that has been identified in main biofluids.

Ethyl pyruvate (pyruvic acid, ethyl ester; CAS 617-35-6).

A SR centered at 1130 cm^-1^ was identified as ethyl pyruvate after its direct spectroscopic measurement and comparison with the experiment. The metabolite represents a derivative of another class of acids, namely alpha-keto acids. It has been identified in [3] but is absent in the compendium [4]. The latter could relate to the fact that pyruvic acid is not an end product but rather a source of other metabolites like SCFAs, carbohydrates etc. Pyruvic acid is known as a product of bacterial metabolism [11]. The pyruvate metabolism was found to be the most dysregulated for PCa [12]. As ethyl pyruvate suppresses PCa tumor [13], it could therefore be down-regulated in cancer.

Carbon monoxide (CAS 630-08-0).

Carbon monoxide has a pronounced absorption spectrum centered at 2170 cm^-1^ (Figure S5 in SM) and therefore can be unambiguously identified. This metabolite is an endogenous catabolic metabolite, with strong relation to smokers’ breath [6]. It was identified in the compendium [4] and in HMDB database [3]. A link between carbon monoxide and PCa was already demonstrated [14]. It is known that this molecule influences cellular bioenergetics, different for cancer and normal cells.

Carbon dioxide (CAS 124-38-9).

Similar to carbon monoxide, the molecule has a pronounced absorption spectrum (Figure S6 in SM). We have chosen its absorption structure centered at 1050 cm^-1^. The metabolite is one of the key molecules involved in plenty of biochemical pathways and described in the compendium [4] (breath) and in the database [3] (blood and urine), with many relations to diseases. Carbon dioxide is produced during respiration (i.e. the process that releases energy from glucose). Its down-regulated level detected in the experiment for cancer cases (Figure S6 in SM, column 4 in Table 1) can be explained by the Warburg effect [15]. Namely, the decreased oxidative phosphorylation leads to impairment in CO_2_ levels inside and outside the cancer cell.

In general, the larger the number of pathways in which a metabolite is involved, the more variations among individuals can be expected. This hypothesis correlates with the data collected in Figure S7, Tables 1 and S3.

References to this section

[1] [www.chemicalbook.com/](http://www.chemicalbook.com/SpectrumEN_108-24-7_IR1.html)

[2] Linstrom, J. & Mallard, W.G. NIST Chemistry WebBook, NIST Standard Reference Database Number 69, Eds. <https://webbook.nist.gov/chemistry/>..

[3] Wishart, D. S. et al., HMDB 4.0 — The Human Metabolome Database for 2018, *Nucleic Acids Res.* **46***,* D608-17 (2018).

[4] Costello, de L. B. et al. A review of the volatiles from the healthy human body, *J. Breath Res.* **8**, 014001 (2014).

[5] Rothman, L. S. et al. The HITRAN 2012 molecular spectroscopic database, *JQSRT* **130**, 4-50 (2013).

[6] Maiti, K. S., Lewton, M., Fill, E. & Apolonski, A. "Human beings as islands of stability: Monitoring body states using breath profiles". *Scientific Reports* **9,** 16167 (2019).

[7] Maiti, K. S., Roy, S., Lampe, R. and Apolonski, A. Breath indeed carries significant information about a disease. Potential biomarkers of cerebral palsy. *J. Biophoton*. **13**, e202000125 (2020). https://doi.org/10.1002/jbio.202000125

[8] Lees, G. J. & Jago, G. R. Acetaldehyde: An intermediate in the formation of ethanol from glucose by lactic acid bacteria. *J. Dairy Res*. **43**, 63 (1976).

[9] Hosoi, N., Ozaki, C., Kitamoto, Y. & Ichikawa, Y. Purification and properties of aldehyde dehydrogenase (acylating) from propionibacterium freudenreichii. *J. Ferment. Technol*. **57**, 418-427 (1979).

[10] Banez, L. L. et al. The effect of acute and chronic exposure to acetaldehyde—a mutagenic metabolite of dietary ethanol—on prostate cancer cells: A potential source of race disparity disfavoring black men. *Journal of Clinical Oncology* **30,** 143-143 (2012).

[11] Cook, R.P. Pyruvic acid in bacterial metabolism: With an account of the methods used for the detection and determination of pyruvic acid. *Biochem J*. **24**,1526-1537 (1930). doi:10.1042/bj0241526]

[12] Lima, A.R. *et al.* Identification of a biomarker panel for improvement of prostate cancer diagnosis by volatile metabolic profiling of urine. *Br. J. Cancer* **121,** 857–868 (2019). <https://doi.org/10.1038/s41416-019-0585-4>

[13] Huang, B. et al. [Suppressed epithelial-mesenchymal transition and cancer stem cell properties mediate the anti-cancer effects of ethyl pyruvate via regulation of the AKT/nuclear factor-kappaB pathway in prostate cancer cells](https://doi.org/10.3892%2Fol.2018.8958). *Oncology Letters* **16**, 2271-2278 (2018).

[14] Wegiel, B. at al. Carbon monoxide expedites metabolic exhaustion to inhibit tumor growth. *Cancer research*  **73**, 7009-7021 (2013). DOI: 10.1158/0008-5472.CAN-13-107

[15] Schwartz, L., Supuran, C.T. & Alfarouk, K.O. The Warburg effect and the hallmarks of cancer. *Anticancer Agents Med. Chem.***17**, 164-170 (2017). doi: 10.2174/1871520616666161031143301.

**6. A possible scenario for the AA production [1]** (combined references for sections 6-9).

We consider a chain of two reactions leading to the AA production [1]:

CH_3_CHO (AD) + O_2_ ----> CH_3_COOOH  (peracetic acid) (1) [2]

CH_3_COOOH + CH_3_CHO ----> (CH_3_CO)_2_O (AA) + H_2_O. (2)

AD in the first reaction can be produced as a direct result of [ethanol and/or pyruvate](https://www.sciencedirect.com/topics/medicine-and-dentistry/alcohol-metabolism) transformation occurring in bacteria that populate the urogenital system. We consider acetic acid bacterium and *Propionibacterium* as bacterial candidates having necessary metabolisms for the production of acetaldehyde and acetic acid. Importantly, these bacteria are present in the urogenital tract of healthy and infected individuals [3]. Upon infection by the bacteria, the production of AA in the urogenital system is enhanced [3]. An indication indirectly supporting the bacterial-based mechanism (1) implies an elevated acetic acid level in the patients having urogenital system infection [4].

Reactions (1) and (2) have a reasonable rate at humanbody temperature. Oxygen in reaction (1) can be taken from the blood oxygen in the arterial bloodstream (see a hypothesized transportation scheme in section 9). Exothermic reaction (2) requires efficient cooling. For this purpose, the addition of low-boiling solvents has been found to be of assistance. Methyl and ethyl acetates are favored because they form azeotropic mixtures with water (but not with acetic acid or AA) and hence allow a rapid, continuous separation of the water formed in the reaction.

As AA is highly hydrophilic, only a small amount reaches the ungs via bloodstream, with further excretion. Ab initio calculations show that the half-lifetime of AA in water at 35°C is approximately 2 minutes [5], with its longer value in salt water mimicking blood. This time is enough to reach the lungs from the prostate gland via the venous part of bloodstream, along the way C-A-B in Figure S9. After its generation, AA immediately participates in acetylation either directly (before its hydrolysis) or as the acetyl group (see section 6).

**7. Acetylation and its relation to carcinogenesis.**

Acetylation refers to the process of introducing an acetyl group (CH3CO^+^) into a chemical compound, and AA is one of an acetylating agent reacting with free hydroxyl groups. In this description, AA being a weak compound, can be considered as a source of an acetyl group (Figure S8). An opposite process of


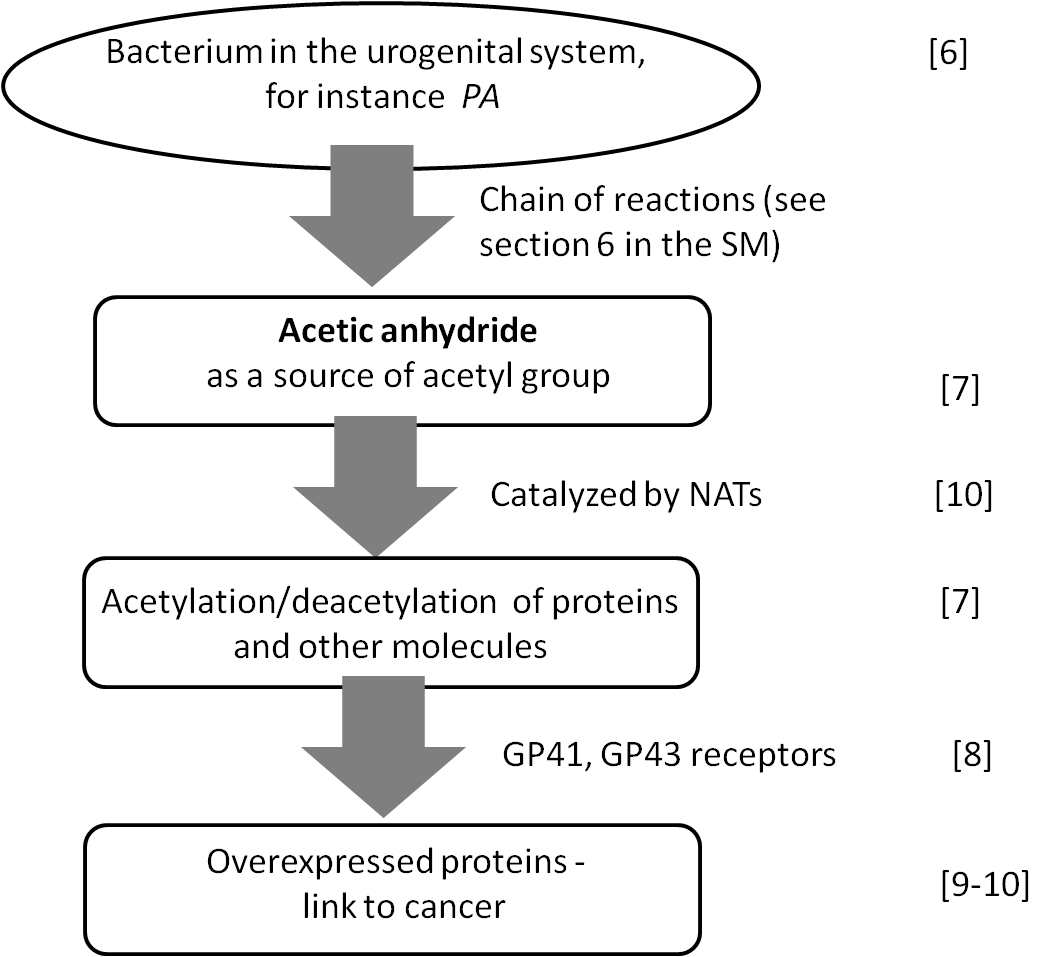


*Figure S8. Involvement of AA and AD (via chain of reactions in section 6) in carcinogenesis.*

deacetylation applied to the same compounds, removes an acetyl group. Acetylation is an important modification of such proteins like histones and p53. Moreover, this process also influences gene expression and metabolism. It was found for some bacteria that most of proteins involved in its central metabolism are acetylated [11]. Specifically, [N-terminal](https://en.wikipedia.org/wiki/N-terminal) acetylation is one of the most common modifications of proteins. This process is catalyzed by a set of enzyme complexes, called the [N-terminal acetyltransferases](https://en.wikipedia.org/wiki/N-terminal_acetyltransferase) (NATs). NATs transfer an acetyl group from [acetyl-coenzyme A](https://en.wikipedia.org/wiki/Acetyl-coenzyme_A) (Ac-CoA) to the α-amino group of the first [amino acid](https://en.wikipedia.org/wiki/Amino_acid) residue of the protein. NATs have been suggested to act as both onco-proteins and tumor suppressors in human cancers, and NAT expression may be increased and decreased in cancer cells [10]. For example, ectopic expression of hNaa10p increased [cell proliferation](https://en.wikipedia.org/wiki/Cell_proliferation) and up regulation of gene involved in cell survival proliferation and [metabolism](https://en.wikipedia.org/wiki/Metabolism). Overexpression of hNaa10p was found in the BC, [breast cancer](https://en.wikipedia.org/wiki/Breast_cancer) and [cervical carcinoma](https://en.wikipedia.org/wiki/Cervical_cancer) [10]. A relation of deacetylation of histone proteins and cancer is solid [7]. Similar to AA, AD is involved in the chain of reactions shown in section 5 and Figure S8.

**8. Bacterial-initiated PCa carcinogenesis. State of the art achievements and speculations.**

There is a growing body of evidence that the human microbiome is linked to cancer [12] and microbial signatures of tumors in blood and tissues have been identified for many types of cancers [13]. Even though their origins are not well understood, they can become a high throughput diagnostic carriers of cancer types including urogenital ones. For example, it is agreed that under some circumstances, colonization of stomach mucose by *Helicobacter pylori* bacterium can lead to the development of gastric cancers [14]. In a similar way, more research papers are focused on the link between *Propionibacterium acnes (PA)* and prostatitis, benign prostatic hyperplasia and malignant prostate cancer [15-17]. The reason for such interest is based on the fact that the exact pathogenic mechanism of the disease is still unknown. Two models developed recently demonstrate that *PA* may serve as a contributing agent to prostatic inflammation [18-19]. *PA* colonized urethral flora is considered as a significant contributing factor in the PCa development since 2003 [6]. Several arguments support this point of view: 1) *PA* leads to an increased cell proliferation as well as cytokine and chemokine secretion in infected prostate cells [16]; 2) *PA* is the most common microorganism in prostate tissue obtained from men with PCa [20] and was isolated from 80% of males [21]; 3) *PA* fits the expected profile of the infectious agent in question, being slow growing with low virulence, difficult for the host to eradicate, capable of causing considerable inflammation and having proven ability to act in synergy with other cofactors [15]; 4) no association between *PA* and tumor stage, grade, the Gleason score [22] and PSA level [13]. It could be explained by a critical infection level that initiates carcinogenesis; 5) men with *PA* had a statistically significant more than 4-fold increase the likelihood of PCa compared to men without the bacterium [13]; 6) patients receiving more than 24 months of medical treatment for *PA* had a reduced risk of PCa [22]; 7) no cancer trigger is necessary: its slow development is defined by the aggregate effect of natural malfunctional factors (see item 1) increasing with age; 8) *PA* was also found in the benign prostatic hyperplasia [23] and in bladder cancer tissue [24] of the joint urinary system. It is still debatable whether *PA* is a disease-causing agent or common contaminant [24].

**9. A transportation scheme.**

A hypothesized transportation scheme shown in Figure S9, is capable to explain the elevated level of metabolites in breath (column 4 of Table 1). Down regulated metabolites in Table 1 can be attributed to other effects of carcinogenesis having no direct link to bacterial infection. In the scenario that we hypothesize, in addition to the normal cycle of the SCFA, AA and AD production in the gut [26] that is illustrated by channel 1, a second channel appears in the infected urogenital system. The urine-blood


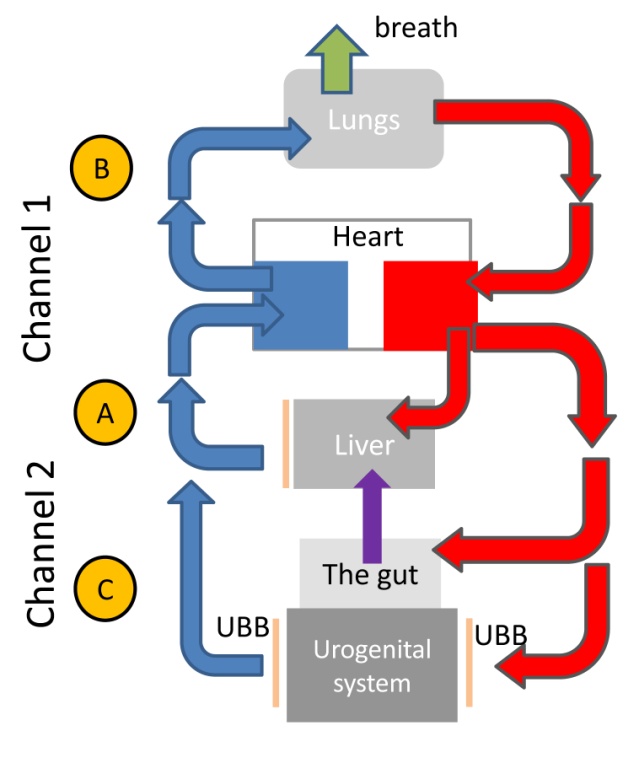


Figure S9. A working model of the origin and transportation of AA, AD and derivatives of SCFAs. Breath of a cancer patient of the urogenital system via input B has contributions from two channels: channel 1 (A-B) and channel 2 (C-A-B), whereas breath of a healthy volunteer mostly contains the metabolites from channel 1.

barrier (UBB) should be considered seriously for supporting a possibility of the metabolite transport from the urogenital system to excreted air. It is known that UBB is one of the strongest in the body [27-28]. To support our basic concept, this barrier should be partially transparent in the direction from blood into the urogenital system for bacteria (to provide initial infection) and be (semi-)transparent for small metabolites in the opposite direction. Such unusual permeability is feasible only in case if this barrier is active, providing selective and non-unidirectional transport. Identification of i) a distinct location of the bacteria within the organ tissue, ii) tubular reabsorption in kidney that connects the urine and blood and iii) possible pathways of the metabolites from there, would help to improve the transportation scheme.

As the *PA* metabolism implies the production of SCFAs and ethanol [29], the following SCFA esterification in the prostate gland by the same bacterium can be considered as the most efficient first stage of the transportation scheme related to PCa. The esters in comparison to the parent SCFAs demonstrate high penetration rate into the bloodstream from the urogenital system and efficient propagation to the lungs with further efficient release in gas phase via alveoli.

**10. Distortion of the baseline.**

**
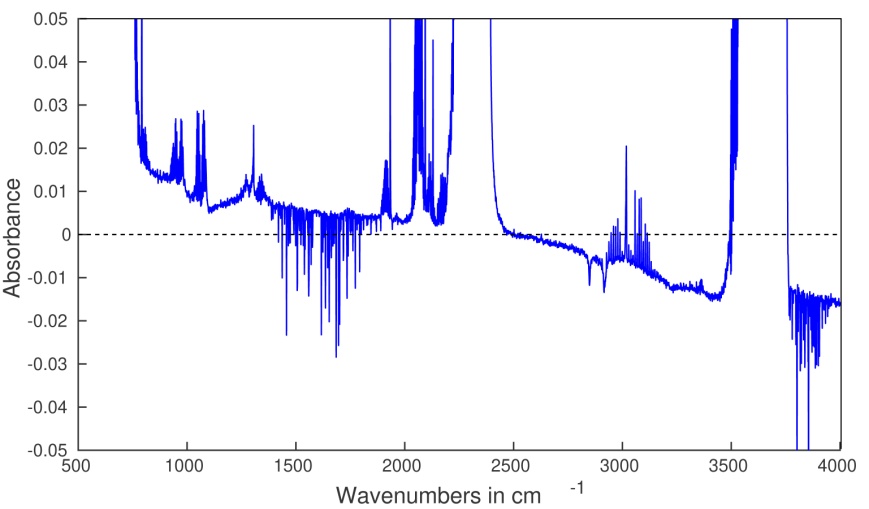
**

Figure S10. An experimental absorption curve of a breath sample in the entire range 500-4000 cm^-1^. There are strong absorption structures linked to CO_2_, water, methane.

**11. Minimum concentration detectable due to noise.**

Experimental dependencies of the 1/noise that defines minimum detectable concentration, on the number of scans and spectral resolution are shown on Figures S11-12.

For the spectrometer, a noise level of

noise = 2.5 x 10^-4^ /(res x nsc)^1/2^ (3),

was measured, where res is the spectral resolution in cm^-1^ and nsc is the number of scans. At a resolution of 1 cm^-1^ a scan takes about 1 s. Applying a signal to noise ratio of three, a minimum detectable concentration in ppm (parts per million) is given by

c_min_ = 3 x noise /[absorbance (ppm m) x L] (4),

where L is the absorption path length in m. For a strongest absorption line with an absorbance of 5 x 10^-4^ (ppm/m), at a resolution of 1 cm^-1^ and the 4 m path length of the multipass cell, equation (4) gives a minimum concentration of 375 ppb nsc^-1/2^. With 100 scans (that take about 100 s at 1 cm^-1^) the minimum detectable concentration is about 40 ppb.

It has to be noted that the minimum detectable concentration depends on the SR. For example, water that has “noisy” absorption bands (Figure S10) leads to lower spectrometer sensitivity or, alternatively, to higher minimum detectable concentration.

Figure S11. Measured 1/noise vs square root of number of scans.

Figure S12. Measured 1/noise vs spectral resolution in cm^-1^.

**12. 3D vibration of AA linked to SR 1005 cm^-1^.**

See file “3D vibration of AA at 1005 cm-1.avi”

**13. References**

[1] Ullmann's Encyclopedia of Industrial Chemistry, 6^th^ edition, Wiley-VCH, 2003. ISBN: 3527303855, 9783527303854. [doi](https://en.wikipedia.org/wiki/Doi_%28identifier%29):[10.1002/14356007](https://doi.org/10.1002%2F14356007.a15_077)

[2] British Patent 653,942 and United States Patent 2,575,- 159.

[3] Hansen, S., Perry, T. L., Lesk, D. & Gibson, L. [Urinary bacteria: potential source of some organic acidurias](https://www.sciencedirect.com/science/article/pii/0009898172903002). [Clinica Chimica Acta](https://www.sciencedirect.com/science/journal/00098981) [**39**](https://www.sciencedirect.com/science/journal/00098981/39/1)**,** 71-74 (1972).

[4] Sison, R. C. et al. Diagnostic Significance of Urine Acetic Acid Level Among Pregnant Women with Bacterial Urinary Tract Infection, American Journal of Clinical Pathology **144**, Issue suppl_2, A216 (2015).

[5] Wilson, P. Personal communication with, BP Chemicals, 1998.

[6] Nelson, W. G., De Marzo, A. M. & Isaacs, W. B. Prostate cancer. *N. Eng.l J. Med*. **349**, 366–381 (2003).

[7] Glozak, M. A. & Seto, E. Histone deacetylases and cancer. *Oncogene* **26**, 5420-5432 (2007).

[8] Szekeres, C. et al. Expression of short chain fatty acid receptors in human prostate cancer: Role in tumor cell survival and motility. *Cancer Epidemiol. Biomarkers Prev*. 2006 **15** (12 Supplement) B73.

[9] Uhlén, M. et al. Tissue-based map of the human proteome. *Science* **347**(6220), 1260419 (2015).

[10] Kalvik, T. V. & Arnesen, T. Protein N-terminal acetyltransferases in cancer. [Oncogene](https://www.nature.com/onc) **32**, 269–276 (2013).

[11] Zhao, S. et al. [Regulation of cellular metabolism by protein lysine acetylation.](https://www.ncbi.nlm.nih.gov/pmc/articles/PMC3232675)  Science **327,** 1000–1004 (2010).

[12] Goodman, B. & Gardner, H. The microbiome and cancer. *J. Pathol*. **244**, 667-676 (2018).

[13] Poore, G. D. et al. Microbiome analyses of blood and tissues suggest cancer diagnostic approach. *Nature* **579** (7800), 567-574 (2020).

[14] Peek, R. M. & Blaser, M. J. Helicobacter pylori and gastrointestinal tract adenocarcinomas. *Nat. Rev. Cancer* **2,** 28–37 (2002).

[15] Shannon, B. A., Garrett, K. L. & Cohen, R. J. Links between *Propionibacterium acnes* and prostate cancer*. Future Oncology* **2***,* 225-232 (2006)*.*

[16] Davidsson, S. et al. Frequency and typing of Propionibacterium acnes in prostate tissue obtained from men with and without prostate cancer. *Infectious agents and cancer* **11,** 26 (2016).

[17] Bae, Y. et al. Intracellular *Propionibacterium acnes* infection in glandular epithelium and stromal macrophages of the prostate with or without cancer. *PLOS one* **9**, e9032 (2014).

[18] Shinohara, D.B. et al. A mouse model of chronic prostatic inflammation using a human prostate cancer-derived isolate of Propionibacterium acnes. *Prostate* **73**(9), 1007–1015(2013).

[19] Olsson, J. et al. Chronic prostatic infection and inflammation by Propionibacterium acnes in a rat prostate infection model. *PLoS One* **7**(12), e51434 (2012).

[20] Cohen, R.J. et al. *Propionibacterium acnes* associated with inflammation in radical prostatectomy specimens: a possible link to cancer evolution? *J. Urol*. **173**(6), 1969–74 (2005).

[21] Shannon, B. A., Cohen, R.J. & Garrett, K. L. Polymerase chain reaction-based identification of *Propionibacterium acnes* types isolated from the male urinary tract: evaluation of adolescents, normal adults and men with prostatic pathology. *BJU Int.* **98**, 388-392 (2006).

[22] Giles, G. G. et al. Early growth, adult body size and prostate cancer risk. *Int. J. Cancer* **103**, 241–245 (2003).

[23] Nickel, J. C., Downey, J., Young, I. & Boag, S. Asymptomatic inflammation and/or infection in benign prostatic hyperplasia. *BJU Int.* **84,** 976–981 (1999).

[24] Mollerup, S. et al. *Propionibacterium acnes*: disease-causing agent or common contaminant? Detection in diverse patient samples by next-generation sequencing. *J. Clin. Microbiology* **54,** 980-987 (2016).

[25] Maiti, K. S., Lewton, M., Fill, E. & Apolonski, A. Human beings as islands of stability: Monitoring body states using breath profiles. *Scientific Reports* **9,** 16167 (2019).

[26] Besten, G. et al. The role of short-chain fatty acids in the interplay between diet, gut microbiota, and host energy metabolism. *J. Lip. Res.* **54**, 2325-2339 (2013).

[27] Wu, X-R. et al. Uroplakins in urothelial biology, function and disease. *Kidney International* **75**, 1153-1165 (2009).

[28] Kreft, M. E. et al. Formation and maintenance of blood-urine barrier in urothelium. *Protoplasma* **246**, 3-14 (2010).

[29] Shu, M. et al. Fermentation of *Propionibacterium acnes*, a commensal bacterium in the human skin microbiome, as skin probiotics against methicillin-resistant staphylococcus aureus. *PLOS One* **8**, e55380 (2013).
